# miR-143 and miR-145 disrupt the cervical epithelial barrier through dysregulation of cell adhesion, apoptosis and proliferation

**DOI:** 10.1038/s41598-017-03217-7

**Published:** 2017-06-08

**Authors:** Lauren Anton, Ann DeVine, Luz-Jeannette Sierra, Amy G. Brown, Michal A. Elovitz

**Affiliations:** Maternal and Child Health Research Center, Department of Obstetrics and Gynecology, Perelman School of Medicine at the University of Pennsylvania, Philadelphia, PA 19104 USA

## Abstract

Molecular mechanisms regulating preterm birth (PTB)-associated cervical remodeling remain unclear. Prior work demonstrated an altered miRNA profile, with significant increases in miR-143 and miR-145, in cervical cells of women destined to have a PTB. The study objective was to determine the effect of miR-143 and miR-145 on the cervical epithelial barrier and to elucidate the mechanisms by which these miRNAs modify cervical epithelial cell function. Ectocervical and endocervical cells transfected with miR-negative control, miR-143 or miR-145 were used in cell permeability and flow cytometry assays for apoptosis and proliferation. miR-143 and miR-145 target genes associated with cell adhesion, apoptosis and proliferation were measured. Epithelial cell permeability was increased in miR-143 and miR-145 transfected cervical epithelial cells. Cell adhesion genes, JAM-A and FSCN1, were downregulated with overexpression of miR-143 and miR-145. miR-143 and miR-145 transfection decreased cervical cell number by increasing apoptosis and decreasing cell proliferation through initiation of cell cycle arrest. Apoptosis genes, BCL2 and BIRC5, and proliferation genes, CDK1 and CCND2, were repressed by miR-143 and miR-145. These findings suggest that miR-143 and miR-145 play a significant role in cervical epithelial barrier breakdown through diverse mechanisms and could contribute to premature cervical remodeling associated with PTB.

## Introduction

In the United States in 2015, 9.6 percent of all live births were delivered preterm^1^. While this number has been slowly declining since reaching a peak at 12.8 percent in 2006^1^, preterm birth remains the leading cause of perinatal morbidity and mortality in developed countries. Indeed, preterm birth results in approximately 26 billion dollars a year in healthcare costs. Importantly, ex-preterm children are at risk for multiple adverse outcomes including a spectrum of neurobehavioral disorders. While the true societal, medical and economic impact of preterm birth cannot be fully estimated, it is clear that preventing preterm birth would be of great medical and societal importance. Yet, despite the potential impact understanding preterm birth could have on preventing this adverse outcome, the pathophysiological and molecular mechanisms leading to preterm birth still remain unclear and, consequently, effective clinical therapies and interventions for preterm delivery remain extremely limited.

Previous theories attempting to ascribe mechanisms to spontaneous preterm birth have primarily focused on the early initiation of uterine contractions due to a myriad of factors including inflammation^2, 3^. The stimulation of uterine contractions, acting as the primary step in preterm birth, is followed by cervical remodeling and early delivery. While uterine contractions undoubtedly contribute to the progression of preterm birth, we have previously suggested that premature cervical remodeling may be the primary, if not, initiating step in the pathogenesis of spontaneous preterm birth^4–7^. Cervical remodeling is a complex process that begins before the onset of labor and is divided loosely into four phases termed softening, ripening, dilation and postpartum repair^8^. As the cervix is made up of two cellular compartments, 1) stromal tissue which includes smooth muscle, immune and fibroblast cells as well as many extracellular matrix (ECM) components including collagen, hyaluronan and proteogylcans and 2) an epithelial layer lining the cervical canal, each of these phases requires intricate molecular and biochemical communication between the different cell types.

Previous studies by our group and others suggest that compromise of the cervical epithelial barrier promotes cervical remodeling and contributes significantly to the pathogenesis of preterm birth^9–11^. Epithelial cells within the cervicovaginal space must be tightly regulated during pregnancy as they play an integral role in cervical remodeling and growth. Cervical epithelial cells line the cervical lumen creating a barrier to protect the cervical stroma from the invasion of microbes and to regulate paracellular transport through the apical junctional complex present on the epithelial cell membrane. The apical junctional complex regulates cell-cell adhesion, paracellular permeability, and cell polarity and is made up of both tight junction and adherens junction proteins^12^. Tight junctions, made up mostly of the claudin family of proteins^13^, and the adherens junctions, made up mostly of the cadherin family of proteins^14^, regulate the “tightness” of the epithelial cells to each other. Therefore, changes in the composition of the tight and/or adherens junctions can alter the cervical epithelial barrier significantly. In order to maintain the integrity of the cervical epithelial barrier during gestation, cervical epithelial cells also undergo a marked increase in growth and proliferation. Consequently, alterations in epithelial cell number can have a significant impact on barrier function.

While the mechanisms regulating cervical remodeling remain largely unknown, there are many factors that may have the ability to alter the cervical epithelial barrier and, hence, initiate cervical remodeling including alterations in inflammation and infection^9, 15^, biomechanical properties of the cervix^16–18^, microRNAs (miRNAs)^19, 20^ and the cervicovaginal microbiome^21^ and metabolome^22^. In a previous study, we investigated the expression of miRNAs in a cohort of women at high risk for preterm birth^20^. We showed the presence of an altered miRNA profile (99 differentially expressed miRNAs) in the cervicovaginal space weeks, if not months, prior to the initiation of spontaneous preterm birth. We identified two specific miRNAs, miR-143 and miR-145, that were significantly increased in the cervix of women destined to have a preterm birth. miRNAs are short, about 22 nucleotides in length, highly conserved single-stranded RNA molecules that play a critical role in post transcriptional gene regulation. One miRNA has the ability to interact with hundreds of messenger RNAs (mRNA) through matching of the miRNA seed sequence with the 3′ untranslated regions (3′UTR) of specific mRNA targets to negatively affect mRNA stability and translation. Therefore, miRNAs have the ability to regulate large gene networks and have been shown to play a significant role in almost every disease state including cancer and cardiovascular disease^23, 24^ among many others. The increased expression of miR-143 and miR-145 in cervical cells from women destined for a preterm birth suggested that these miRNAs have the ability to contribute significantly to cervical remodeling.

The objective of this study was to determine the effect of miR-143 and miR-145 on the integrity of the cervical epithelial barrier and to elucidate the mechanisms by which these miRNAs modify cervical epithelial cell function. For this study, we investigated the effects of miR-143 and miR-145 on cervical epithelial cell permeability to determine if these miRNAs have the ability to alter the cervical epithelial cell barrier. Additionally, we focused on the molecular mechanisms contributing to the breakdown of the cervical epithelial cell barrier by investigating the effects of increased miR-143 and miR-145 expression on cell adhesion and growth. Therefore, we hypothesize that increased expression of miR-143 and miR-145 disrupts the integrity of the cervical epithelial barrier through regulation of cell adhesion, apoptosis and cell proliferation which initiates premature cervical remodeling resulting in early delivery.

## Results

### miR-143 and miR-145 increase ectocervical and endocervical epithelial cell permeability

**Figure 1:**
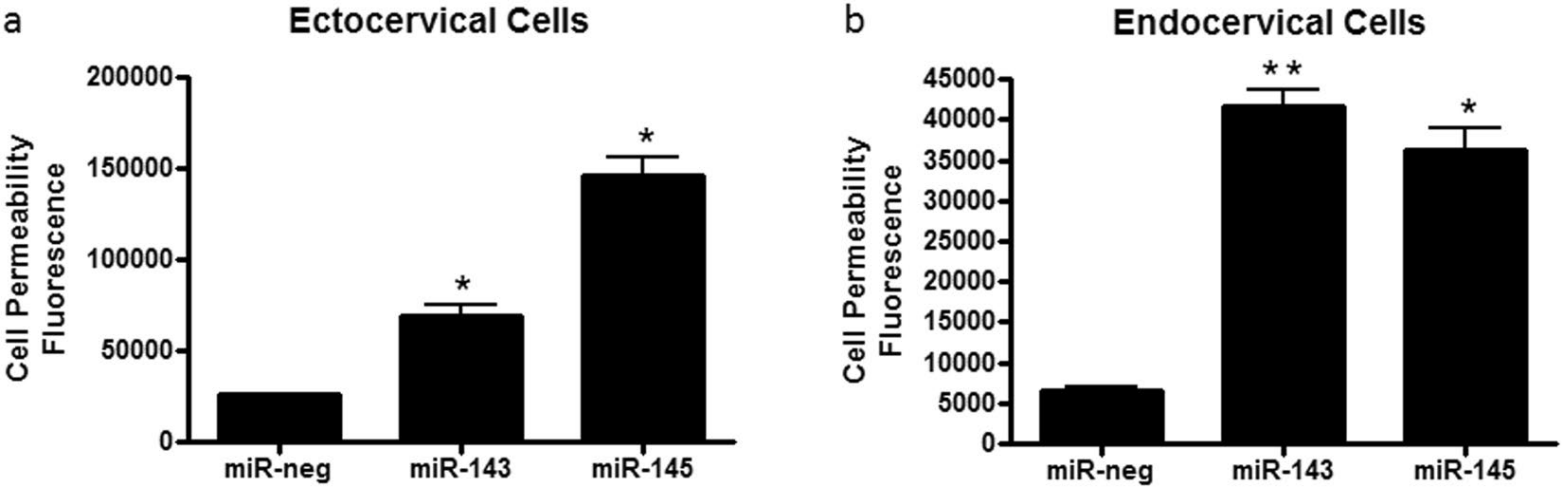
Epithelial cell permeability is altered in ectocervical and endocervical cells transfected with miR-143 and miR-145 mimics. Epithelial cell permeability was significantly increased in ectocervical cells (**a**) and endocervical cells (**b**) overexpressing miR-143 and miR-145 when compared to miR-negative (miR-neg) control. Cell permeability is expressed as fluorescence OD measurements from a fluorescent plate reader and is indicative of the movement of FITC-dextran from the top to the bottom insert of a transwell chamber system. Values are mean ± SEM. *p < 0.001, **p < 0.0001.

As we have previously identified miR-143 and miR-145 as being significantly increased in the cervicovaginal space in high risk women months prior to delivering preterm^20^, we wanted to determine if these specific miRNAs have an effect on cervical cell function. Therefore, we focused on epithelial cell permeability, as decreased epithelial tight junctions and increased water influx are primary events in cervical ripening and remodeling^25^. Ectocervical (Fig. 1a) and endocervical (Fig. 1b) cells transfected with miR-143 (n = 3, ecto: p = 0.0016, endo: p < 0.0001) and miR-145 (n = 3, ecto: p = 0.0003, endo: p = 0.0004) had a significant increase in epithelial cell permeability (when compared to those transfected with miR-negative control) as evidenced by a significant increase in the amount of FITC dextran present in the bottom well of the transwell inserts. These results suggest that increased expression of these miRNAs contribute to the breakdown of the cervical epithelial cell barrier.

### Predicted gene targets of miR-143 and miR-145

In order to determine the specific genes that might be mechanistically involved in alterations of the cervical epithelial barrier, we focused on known or predicted downstream targets of miR-143 and miR-145 that regulate epithelial cell adhesion and cell number including apoptosis and cell proliferation. Using TargetScan^26^, an online software program that predicts biological targets of miRNAs by searching for the presence of 8mer, 7mer, and 6mer sites that match the seed region of a miRNA of interest, we identified several target genes known to be involved in epithelial tight junction formation and cell adhesion including junctional adhesion molecule-A (JAM-A, F11R) and Fascin1 (FSCN1). Additionally, we chose to focus on targets involved in inhibiting the intrinsic apoptosis pathway including B-cell lymphoma 2 (BCL2) and baculoviral inhibitor of apoptosis repeat-containing 5 (BIRC5, Survivin) as well as genes known to play a role in cell cycle progression such as cyclin dependent kinase 1 (CDK1) and cyclin D2 (CCND2). The seed region sequence for miR-143 and miR-145, their predicted genes of interest and their corresponding target sequences are shown in Tables 1 and 2, respectively. A full list of all miR-143 and miR-145 target genes investigated as part of this study and their expression in ectocervical and endocervical cells can be found in Supplementary Table S1.

**Table 1:** Predicted Target Genes of miR-143.

	Position in 3′UTR of target gene	Predicted consequential pairing of target region (5′-3′)
miR-143		3′- CUCGAUGUCACGA**AGUAGAGU**
JAM-A	Not a predicted target	
FSCN1	860–866	CCUCCCCAGGGGGUG**CAUCUCA**G
BCL2	4520–4527	CAAUCAUGAAAUAUG**CAUCUCA**C
BIRC5	1102–1108	GGAAACCUCUGGAGG**UCAUCUC**G
CDK1	1789–1796	UACUGAAUUGUGUCC**UCAUCUCA**
CCND2	2495–2501	ACCUUGUGUUUAGGA**UCAUCUC**U

**Table 2:** Predicted Target Genes of miR-145.

	Position in 3′UTR of target gene	Predicted consequential pairing of target region (5′-3′)
miR-145		3′- UCCCUAAGGACCCU**UUUGACCU**G
JAM-A	692–698	CUCUAAAGAAAAGAA**AACUGGA**G
FSCN1	116–123	CCCCCUUGCCUUUCA**AACUGGA**A
BCL2	Not a predicted target	
BIRC5	2000–2006	GAGACCAGCAAGCC**AAACUGGA**G
CDK1	Not a predicted target	
CCND2	1855–1861	AUAUGAGUUCUUCGU**ACUGGAA**A

### miR-143 and miR-145 decrease cell adhesion

**Figure 2:**
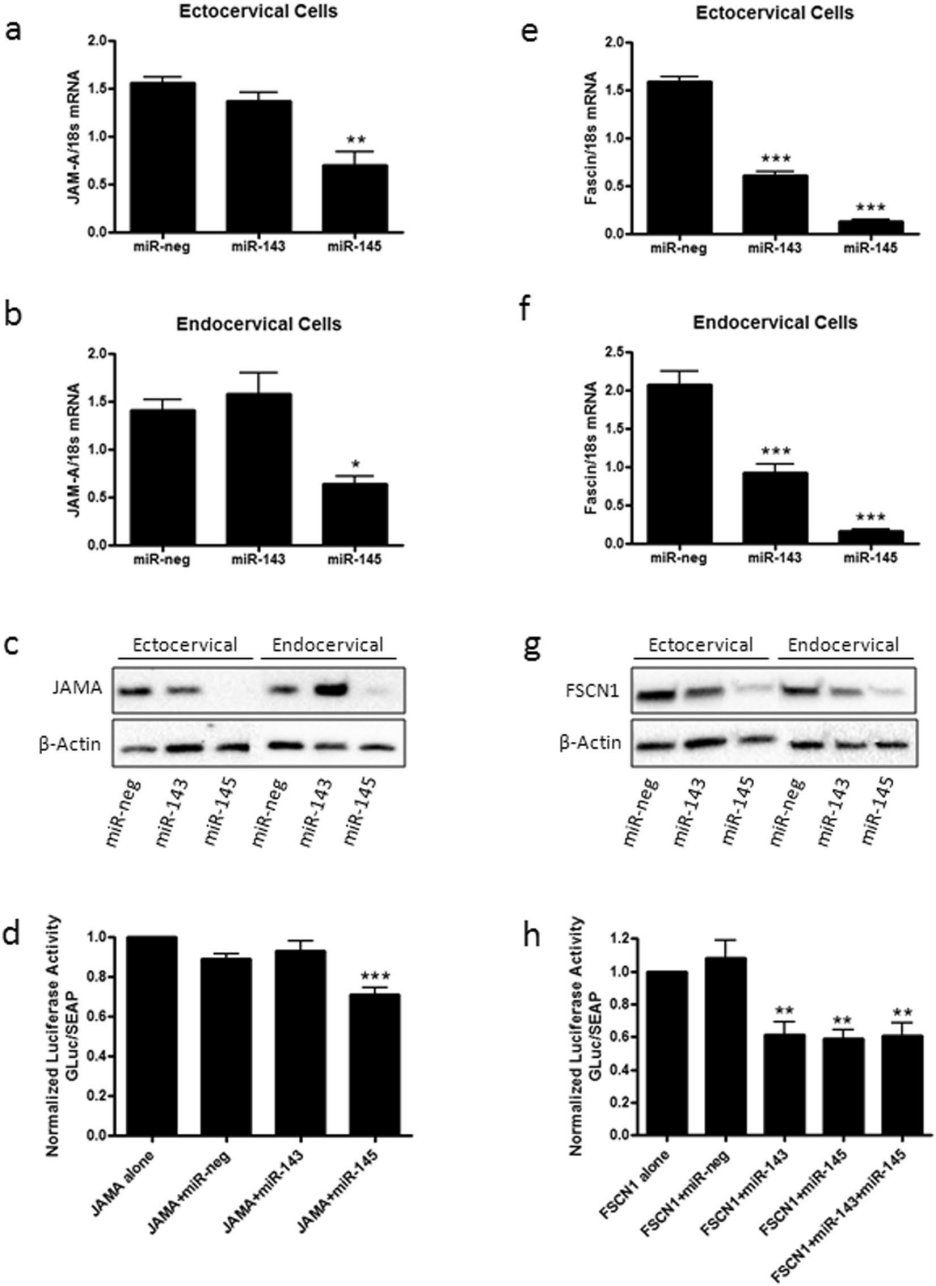
miR-143 and miR-145 inhibit adhesion gene expression in ectocervical and endocervical cells. Ectocervical and endocervical cells were transfected with miR-negative control (miR-neg), miR-143 and miR-145 mimics and expression of downstream target genes were measured by QPCR and western blot. Junctional adhesion molecule-A (JAM-A), a member of the adherens junction complex, was significantly decreased by overexpression of miR-145 but not miR-143 in both ectocervical (**a**) and endocervical cells (**b**). JAM-A protein expression was reduced by miR-145 in ectocervical and endocervical cells (**c**). Fascin-1 (FSCN1), an actin bundling protein, was significantly repressed in both miR-143 and miR-145 transfected ectocervical (**e**) and endocervical (**f**) cells. FSCN1 protein expression was reduced by miR-143 and miR-145 in both cell types (**g**). 3′UTR luciferase reporter assays in HEK293T cells verified that JAM-A is a direct target of miR-145 (**d**) and FSCN1 is a direct target of both miR-143 and miR-145 (**h**). 3′UTR assay results are expressed as a ratio of Gaussia Luciferase (GLuc) activity over Secreted Alkaline Phosphatase (SEAP) which has been normalized to cells transfected with the plasmid alone. Values are mean ± SEM. *p < 0.05, **p < 0.01, ***p < 0.001.

**Figure 3:**
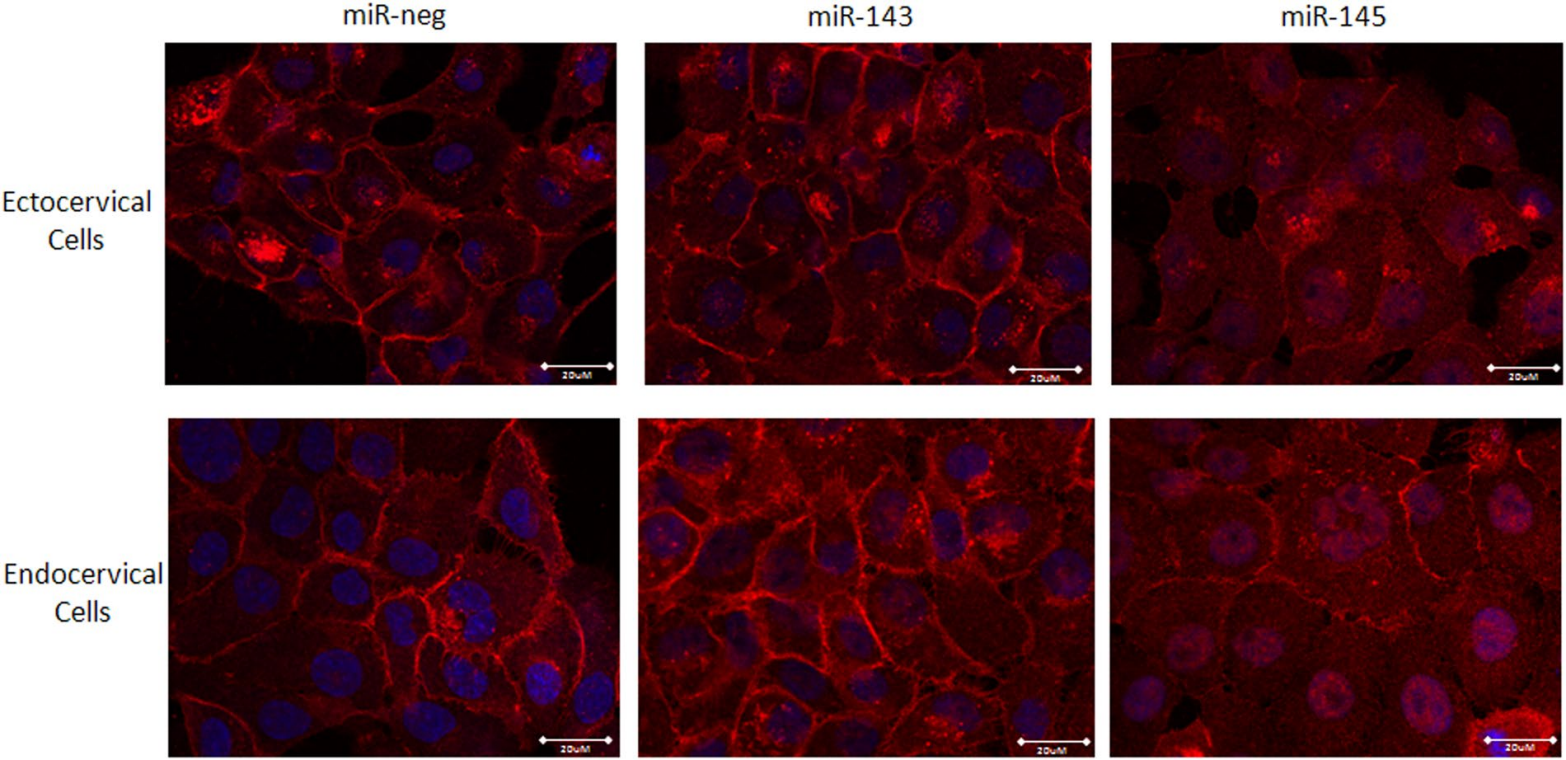
miR-145 inhibits JAM-A expression on the epithelial cell membrane of ectocervical and endocervical cells. Ectocervical and endocervical cells transfected to overexpress miR-143 and miR-145 were stained for JAM-A (red) and DAPI (cell nucleus, blue). JAM-A protein expression was reduced in both ectocervical and endocervical cells transfected with miR-145 but not miR-143 indicating that JAM-A is a direct target of miR-145.

JAM-A, a predicted target of miR-145, was significantly decreased in both ectocervical (Fig. 2a) and endocervical (Fig. 2b) cells transfected with miR-145 (n = 3, ecto: p < 0.01, endo: p < 0.05) but not miR-143. JAM-A protein expression (Fig. 2c) was similarly decreased in ectocervical and endocervical cells transfected with miR-145 but not miR-143. The JAM-A 3′UTR reporter assay (Fig. 2d), which shows repressed GLuc activity in the presence of a specific miRNA target, showed a significant decrease in GLuc expression in the presence of miR-145 when compared to the JAM-A plasmid alone (n = 9, p < 0.001). No changes in GLuc expression were seen in the presence of exogenous miR-neg control or miR-143 indicating that JAM-A is a direct target of miR-145. Staining of ectocervical and endocervical cells provide further evidence that transfected cells have the ability to form a monolayer and that miR-145 but not miR-143 represses JAM-A expression localized at the epithelial cell membrane (Fig. 3). Additional experiments showed a significant decrease in FSCN1 expression after transfection of ectocervical (Fig. 2e) and endocervical (Fig. 2f) cells with both miR-143(n = 3, ecto: p < 0.001, endo: p < 0.01) and miR-145 (n = 3, ecto: p < 0.001, endo: p < 0.001). FSCN1 is an actin bundling protein that regulates cytoskeletal structures for the maintenance of cell adhesion in epithelial cells and is a predicted target for both miR-143 and miR-145. FSCN1 protein expression was reduced after ectocervical and endocervical cell transfection with both miR-143 and miR-145 (Fig. 2g). The FSCN1 3′UTR assay (Fig. 2h) showed repressed GLuc activity in the presence of both miR-143 (n = 6, p < 0.01) and miR-145 (n = 6, p < 0.01) alone and in combination (n = 6, p < 0.01) but not with miR-negative control. These results confirmed that miR-143 and miR-145 specifically target FSCN1 in ectocervical and endocervical cells.

### miR-143 and miR-145 decrease ectocervical and endocervical cell number

**Figure 4:**
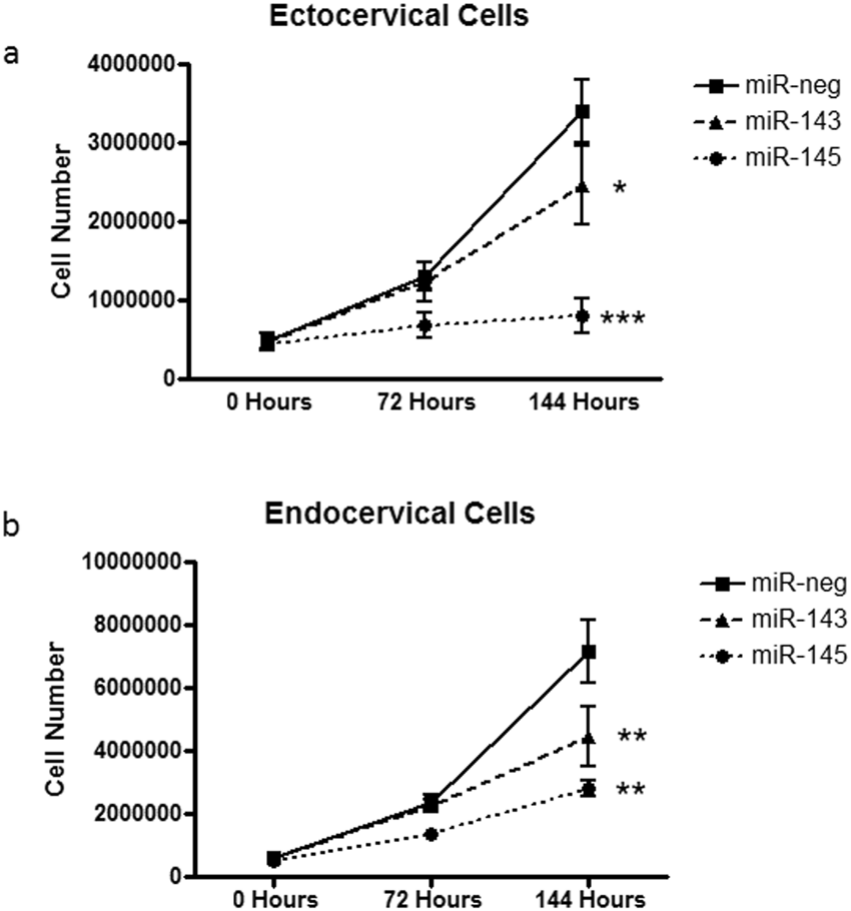
Ectocervical and endocervical cell number is decreased by miR-143 and miR-145. Ectocervical and endocervical cells transfected to overexpress miR-143 and miR-145 were counted at 0, 72 and 144 hours after transfection. The total number of ectocervical (**a**) and endocervical (**b**) cells were significantly decreased in both miR-143 and miR-145 transfected cells when compared to the miR-negative transfected cells after 144 hours of growth. Values are mean ± SEM. *p < 0.05, **p < 0.001, ***p < 0.0001.

When ectocervical (Fig. 4a, n = 3, p = 0.0003) and endocervical epithelial cells (Fig. 4b, n = 3, p = 0.0002) were transfected with either miR-143 or miR-145 there was a significant decrease in cell number over time (ecto: p < 0.0001, endo: p < 0.0001). While the largest decrease in cell number was seen in ectocervical (p < 0.0001) and endocervical (p < 0.001) cells transfected with miR-145 for 144 hours, there was also a decrease in both cell types transfected with miR-143 (ecto: p < 0.05, endo: p < 0.001) when compared to those transfected with miR-neg control. This effect was independent of the transfection reagent (Lipofectamine exposure) as we did not see any effect on cell number when transfecting the miR-negative control into these cells (compared to non-transfected cells, data not shown).

### miR-143 and miR-145 promote apoptosis

**Figure 5:**
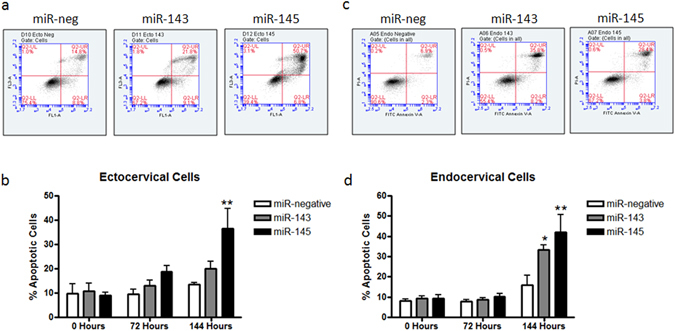
miR-143 and miR-145 increased ectocervical and endocervical cell apoptosis. Flow cytometry based annexin V/PI apoptosis assays were performed on ectocervical and endocervical cells transfected with miR-negative control (miR-neg), miR-143 or miR-145 at 0, 72 and 144 hours after transfection. Representative histograms showing the results of the apoptosis assay are depicted for each miRNA at 144 hours in ectocervical (**a**) and endocervical (**c**) cells. The results from four independent assays are shown in the bar graphs. In ectocervical cells, miR-145 significantly increased the percentage of apoptotic cells (**b**) while, in endocervical cells, both miR-143 and miR-145 increased the percentage of apoptotic cells (**d**) at 144 hours. Values are mean ± SEM *p < 0.05, **p < 0.001.

After observing a significant decrease in cell number after transfection with miR-143 and miR-145, we investigated the molecular mechanisms contributing to this effect. Preliminary experiments, using an ApoTox-Glo Triplex assay which assesses viability, cytotoxicity and caspase activation in a single sample (described in detail in supplementary methods online), showed that both ectocervical and endocervical cells transfected with miR-143 and miR-145 were undergoing apoptosis (regulated cell death) versus cytotoxicity (Supplementary Fig. S2). Given these results, we performed flow cytometry analysis to definitively determine the percentage of cells undergoing apoptosis (positive for Annexin V/PI). Figure 5a and c show a representative histogram of ectocervical and endocervical cells after transfection with miR-neg, miR-143 and miR-145. Cells in the upper right quadrant of each histogram are positively stained for both Annexin V and propidium iodine and represent cells undergoing apoptosis. While we understand that Annexin V/PI positive cells could also represent dead cells that have not gone through the apoptotic process but have instead bound Annexin V due to disrupted cell membranes, given the results of the Triplex assay (Supplementary Figure 2) and that this assay was done over multiple time points with increasing percentages of Annexin V positive, PI-negative cells it is reasonable to believe that the majority of the double-positive cells were at some point apoptotic. Transfection with miR-145 significantly increased the percent of apoptotic ectocervical cells (Fig. 5b, n = 3, p < 0.001) while both miR-143 (p < 0.05) and miR-145 (p < 0.001) increased apoptosis in endocervical cells (Fig. 5d, n = 3) at the 144 hr time point when compared to cells transfected with miR-neg control.

### miR-143 and miR-145 decrease negative regulators of apoptosis

**Figure 6:**
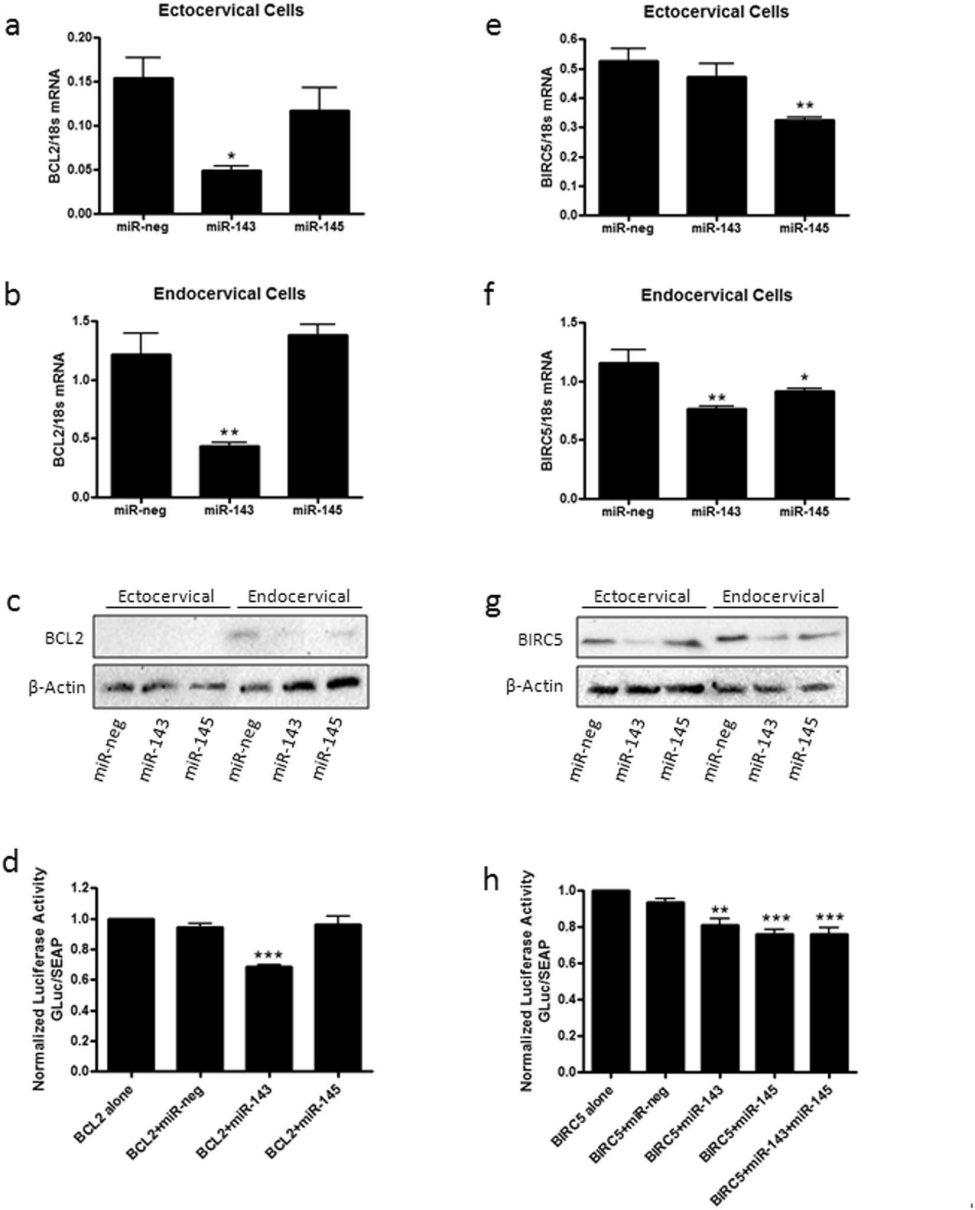
miR-143 and miR-145 repressed apoptosis gene expression. Ectocervical and endocervical cells were transfected with miR-negative control (miR-neg), miR-143 or miR-145 and expression of downstream target genes were measured by QPCR and western blot. BCL2, a negative regulator of apoptosis and a predicted target of miR-143, was significantly decreased with miR-143 but not miR-145 overexpression in ectocervical (**a**) and endocervical cells (**b**). Protein expression of BCL2 was reduced by miR-143 in transfected endocervical cells while BCL2 protein was too low to be detectable in ectocervical cells (**c**). 3′UTR luciferase assays in HEK293T cells confirmed that BCL2 is a direct target of miR-143 (**d**). BIRC5, a predicted target of miR-143 and miR-145, was significantly repressed by miR-145 in ectocervical (**e**) cells and by both miR-143 and miR-145 in endocervical cells (**f**). BIRC5 protein expression was reduced by miR-143 and miR-145 in endocervical cells while BIRC5 protein levels were decreased by miR-143 and unchanged by miR-145 in ectocervical cells (**g**). 3′UTR luciferase assays verified that BIRC5 is a target of miR-143 and miR-145 (**h**). 3′UTR assay results are expressed as a ratio of Gaussia Luciferase (GLuc) activity over Secreted Alkaline Phosphatase (SEAP) which has been normalized to cells transfected with the plasmid alone. Values are mean ± SEM. *p < 0.05, **p < 0.01, ***p < 0.001.

BCL2, a predicted target of miR-143, was significantly decreased in both ectocervical (Fig. 6a) and endocervical (Fig. 6b) cells transfected with miR-143(n = 4, ecto: p < 0.05, endo: p < 0.01) but not miR-145. BCL2 protein expression was reduced in endocervical cells transfected with miR-143 and miR-145. However, in ectocervical cells, in agreement with very low mRNA levels, protein levels were too low to be detected by western blot (Fig. 6c). The BCL2 3′UTR reporter assay (Fig. 6d, n = 9) showed a significant decrease in GLuc expression in the presence of miR-143 (p < 0.001) when compared to the BCL2 plasmid alone. No changes in GLuc expression were seen in the presence of exogenous miR-neg control or miR-145 indicating that BCL2 is a direct target of miR-143. A significant decrease in BIRC5 expression was seen in ectocervical cells (Fig. 6e, n = 4) transfected with miR-145 (p < 0.01). Transfection with both miR-143 (p < 0.01) and miR-145 (p < 0.05) repressed BIRC5 in endocervical cells (Fig. 6f, n = 4). Protein expression of BIRC5 was decreased after transfection of endocervical cells with miR-143 and miR-145. However, in ectocervical cells, there was a reduction in BIRC5 protein expression in miR-143 transfected cells while BIRC5 was unchanged after transfection with miR-145 (Fig. 6g). Decreased BIRC5 3′UTR GLuc activity (Fig. 6h, n = 6) was seen in the presence of exogenous miR-143(p < 0.01), miR-145 (p < 0.001) and both miR-143 and miR-145 co-transfected together (p < 0.001) but not the miR-negative control indicating that BIRC5 is a direct target of both miRNAs.

### miR-143 and miR-145 alter cell cycle progression

**Figure 7:**
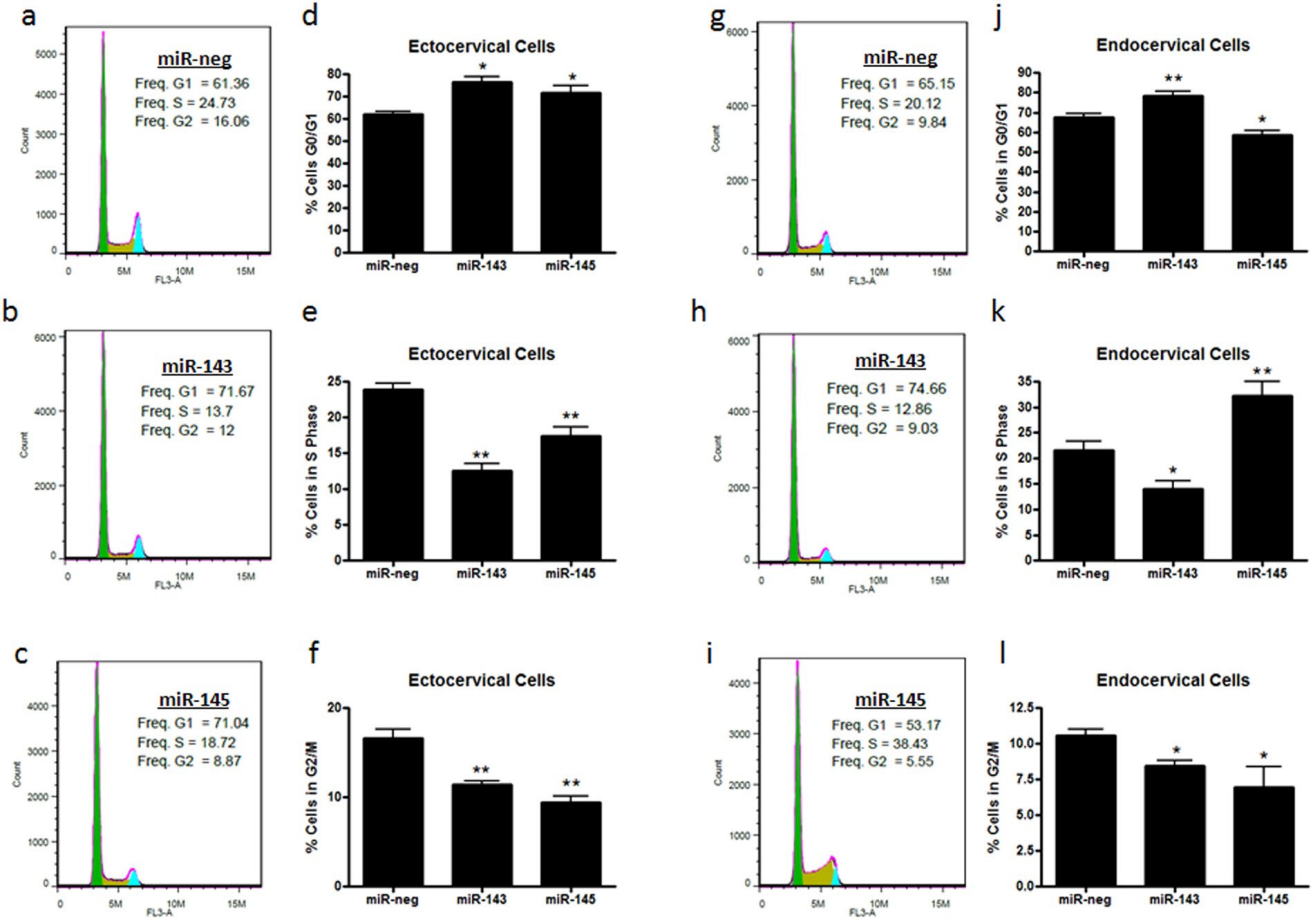
miR-143 and miR-145 overexpression results in cell cycle arrest. Ectocervical and endocervical cells transfected with miR-negative control (miR-neg), miR-143 or miR-145 were used for cell cycle assays by flow cytometry. Representative cell cycle histograms show the percentage of ectocervical and endocervical cells in each phase of the cell cycle after miR-neg (**a**,**g**), miR-143 (**b**,**h**) and miR-145 (**c**,**i**) transfection. Bar graphs show the average (n = 4 independent experiments) percentage of cells in each phase of the cell cycle. miR-143 and miR-145 over expression resulted in cell cycle arrest at G0/G1 (**d**) in ectocervical cells (**d**,**e**,**f**). In endocervical cells (**j**,**k**,**l**), similarly, miR-143 transfection caused cell cycle arrest at G0/G1 (**j**), while, miR-145 transfection resulted in S-phase arrest (**k**). Values are mean ± SEM *p < 0.05, **p < 0.01.

In addition to the role of miR-143 and miR-145 in promoting apoptosis, we also investigated if decreases in cell number could be due to alterations in cell proliferation and growth. Using flow cytometry, we performed standard cell cycle assays to assess if ectocervical and endocervical cells transfected with miR-neg, miR-143 and miR-145 were progressing normally through the three phases of cell growth – G0/G1, synthesis (S-phase) and G2. Representative histograms demonstrating the cell cycle after transfection with miR-neg (Fig. 7a,g), miR-143 (Fig. 7b,h) and miR-145 (Fig. 7c,i) are shown for ectocervical and endocervical cells. Quantification of the percentage of cells in each of the three phases of the cell cycle show a significant increase in ectocervical cells (n = 3) in the G0/G1 phase (Fig. 7d, p = 0.0113) after transfection with miR-143 (p < 0.05) and miR-145 (p < 0.05) when compared with miR-neg. Consistent with this, there is a significant reduction in the percentage of cells in the S-phase (Fig. 7e, miR-143: p < 0.01, miR-145: p < 0.01) and G2 phase (Fig. 7f, miR-143: p < 0.01, miR-145: p < 0.01) indicating cell cycle arrest at G0/G1. A similar effect is seen in endocervical cells (n = 4) transfected with miR-143 at G0/G1 (Fig. 7j, p < 0.01), S-phase (Fig. 7k, p < 0.05) and G2 (Fig. 7l, p < 0.05) but not with miR-145. miR-145 transfection decreased the percentage of cells in G0/G1 (Fig. 7j, p < 0.05) and increased endocervical cells in S-phase (Fig. 7k, p < 0.01) followed by a reduction in cells in G2 (Fig. 7l, p < 0.05) suggesting that miR-145 causes cell cycle arrest in the S phase.

### miR-143 and miR-145 repress cell cycle regulators

**Figure 8:**
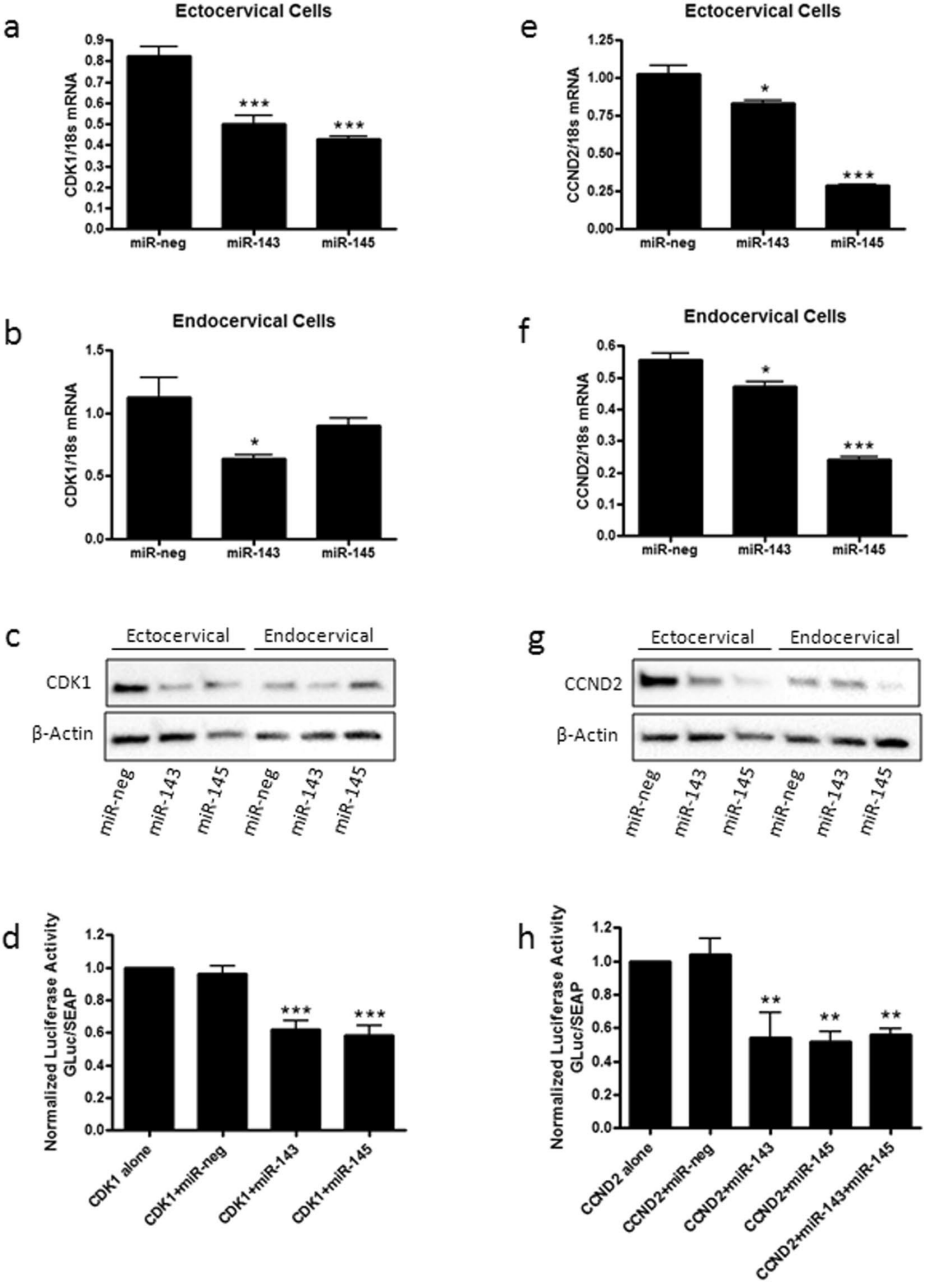
miR-143 and miR-145 repress genes that regulate the cell cycle. Ectocervical and endocervical cells were transfected with miR-negative control (miR-neg), miR-143 or miR-145 and expression of downstream target genes were measured by QPCR and western blot. Cyclin dependent kinase 1(CDK1), was significantly decreased by both miR-143 and miR-145 in ectocervical cells (**a**). In endocervical cells, only miR-143 transfection resulted in a decrease in CDK1 expression (**b**). Protein expression of CDK1 was reduced by miR-143 and miR-145 in ectocervical cells and by miR-143 only in endocervical cells (**c**). 3′UTR luciferase assays confirmed that CDK1 is a direct target of miR-143 and miR-145 (**d**). Cyclin D2 (CCND2) was repressed by overexpression of both miR-143 and miR-145 in ectocervical (**e**) and endocervical (**f**) cells. CCND2 protein expression was reduced in miR-143 and miR-145 transfected ectocervical and endocervical cells (**g**). 3′UTR luciferase assays showed that CCND2 is a direct target of both miR-143 and miR-145 (**h**). 3′UTR assay results are expressed as a ratio of Gaussia Luciferase (GLuc) activity over Secreted Alkaline Phosphatase (SEAP) which has been normalized to cells transfected with the plasmid alone. Values are mean ± SEM. *p < 0.05, **p < 0.01, ***p < 0.001.

CDK1, an integral player in cell cycle progression, is significantly repressed after miR-143 (p < 0.001) and miR-145 (p < 0.001) transfection in ectocervical cells (Fig. 8a, n = 4) and with miR-143 transfection only (p < 0.05) in endocervical cells (Fig. 8b, n = 4). Similar to mRNA transcript levels, CDK1 protein expression was reduced in ectocervical cells transfected with miR-143 and miR-145, while in endocervical cells, CDK1 was decreased in miR-143 transfected cells only (Fig. 8c). The 3′UTR reporter assay for CDK1 showed significant reductions in GLuc activity in the presence of miR-143 and miR-145 indicating that CDK1 is acting as a direct target of miR-143 and miR-145 (Fig. 8d, n = 12). CCND2, regulator of the cell cycle during the G1/S transition, is significantly repressed after transfection of miR-143 (ecto: p < 0.05, endo: p < 0.05) and miR-145 (ecto: p < 0.001, endo: p < 0.001) in ectocervical (Fig. 8e, n = 4) and endocervical cells (Fig. 8f, n = 4). Protein expression of CCND2 was reduced in both miR-143 and miR-145 transfected ectocervical and endocervical cells (Fig. 8g). The CCND2 3′UTR reporter assay expressed decreased GLuc activity in the presence of both miR-143 (p < 0.01) and miR-145 (p < 0.01) alone and in combination (p < 0.01) indicating that CCND2 is a direct target of both miRNAs (Fig. 8h, n = 9).

## Discussion

This study provides novel insight into the molecular mechanisms contributing to cervical remodeling. Additionally, as we have shown in a previous study that miR-143 and miR-145 are significantly increased in the cervical vaginal space of women destined to have a premature delivery^20^, this study suggests that compromise of the cervical epithelial barrier is a possible mechanism for the pathogenesis of preterm birth. First, we have shown that overexpression of miR-143 and miR-145 results in a breakdown of the cervical epithelial barrier in both ectocervical and endocervical cells. Secondly, we have identified multiple molecular pathways and specific gene targets contributing to the increase in epithelial barrier breakdown. Increased expression of miR-143 and miR-145 alters both ectocervical and endocervical cell function by decreasing adherens junction proteins, reducing cell number, activating the intrinsic apoptosis pathway and initiating cell cycle arrest. Additionally, similar results in both ectocervical and endocervical cells indicate that miR-143 and miR-145 have global, and not region-specific, effects on the cervical epithelia. We acknowledge that the expression levels of miR-143 and miR-145 following transfection supersedes the physiological levels seen in our previous study^20^, however, understanding the limitations of *in vitro* work, we believe that our data support the biological plausibility of the role of these miRNAs in cervical epithelial biology. The results of this study support our hypothesis that the pathogenesis of preterm birth is initiated within the cervical vaginal space where alterations in the environment surrounding the cervix can alter cervical function leading to premature cervical remodeling.

During pregnancy, the cervical epithelial cells must maintain a strong barrier in order to protect the upper reproductive tract and fetus from invading pathogens, regulate paracellular transport and maintain fluid balance. However, in the presence of an “unhealthy” cervicovaginal space, characterized by increased inflammation due to a bacterial infection or a dysbiotic microbiome, among other possibilities, the cervical epithelial barrier could become compromised. Once the epithelial barrier is disrupted, the cervical stroma is vulnerable to invasion by pathogenic bacteria, inflammatory mediators or water^25^ which have all been previously associated with the initiation of cervical tissue remodeling. The concept of a compromised epithelial barrier resulting in a disease state is well described in the gut literature^27–30^. In the gastrointestinal tract, commensal bacteria and food-derived antigens directly interact with the gut epithelial barrier, which acts similarly in the cervix, to create a physical and immunological barrier against invading pathogens. Disruption of the gut epithelial barrier results in invasion by commensal and pathogenic bacteria causing recruitment of pro-inflammatory mucosal immune mediators. This detrimental inflammation ultimately leads to inflammatory bowel disease, Crone’s disease and ulcerative colitis. In a previous study, we have shown that the presence of lipopolysaccharide (LPS), a gram negative inflammatory mediator, has the ability to breakdown the cervical epithelial barrier through increased cytokine expression and sECAD release^9^. The results from that study suggested that inflammatory agents, gaining access to the cervix through the vaginal canal, have the ability to compromise the epithelial barrier. Similarly, in this study, we showed that increased expression of miR-143 and miR-145 were able to disrupt the epithelial barrier. Interestingly, we have previously shown that ectocervical and endocervical cells exposed to LPS have increased expression of miR-143 and miR-145 suggesting that inflammation could be an upstream regulator of these miRNAs^20^. While some progress has been made in understanding the role of inflammation in cervical remodeling, the molecular mechanisms regulating this process remain largely unclear. As miR-143 and miR-145 are undoubtedly regulated by both inflammation- dependent and independent mechanisms, it is of interest to determine the molecular mechanisms altered by miR-143 and miR-145 that contribute to the breakdown of the cervical epithelial barrier.

Since cell to cell adhesion is regulated by the presence of both tight junctions and adherens junctions on the epithelial membrane, we first focused on investigating the proteins localized to these two junctional complexes. After doing a search of known or predicted target genes of miR-143 and miR-145 in TargetScan, two proteins in particular were of most interest including JAM-A and Fascin-1 (FSCN1). JAM-A, a predicted target of miR-145, is an integral part of the adherens junction complex, and has been shown to contribute significantly to epithelial cell barrier function^31, 32^. FSCN1, a direct target of both miR-143 and miR-145, is an actin bundling protein that regulates cell adhesion and cellular interactions^33^. In this study, we found a significant reduction in both JAM-A and FSCN1 gene expression in the presence of miR-143 and miR-145 overexpression in ectocervical and endocervical cells suggesting that reductions in JAM-A and FSCN1 contribute to increased epithelial cell permeability. Although no previous studies have been done investigating the effects of miR-143 or miR-145 on the cervical epithelial barrier, miR-145 is known to target JAM-A in endometrial stromal cells^34^ and breast cancer cells^35^ where it inhibited cell proliferation, motility and invasion indicating that cervical cell function could be similarly affected. Similar studies investigating miR-145 on FSCN1 expression in several cancer cell lines (gastric, colorectal, non-small cell lung) have shown significant inhibition in cancer cell phenotypes including metastasis, invasion and proliferation^36–38^. As most research involving miR-143 and miR-145 has been done in the cancer field, no studies have investigated the effects of these miRNAs on cellular adhesion as it relates to cell barrier function. Therefore, this study is the first to associate miR-143 and miR-145 with deficits in the epithelial barrier.

Disruption of the cervical epithelial barrier is associated with alterations in both epithelial cell number and epithelial cell proliferation^39^. As previous studies have shown that miR-143 and miR-145 have significant effects on cancer cell growth^40^, we focused on cervical cell number and miRNA target genes known to affect cell apoptosis and proliferation. In the presence of overexpressed miR-143 and miR-145, we found decreased ectocervical and endocervical cell number as well as increased apoptosis. BCL2, a target of miR-143, and BIRC5, a target of miR-143 and miR-145, are both well studied inhibitors of the intrinsic apoptosis pathway. Increased miR-143 and miR-145 inhibited the expression of BCL2 and BIRC5, consequently, activating the apoptosis pathway. These results indicate that upregulated miR-143 and miR-145 expression within the cervical space could lead to increased apoptosis at the cervical epithelial barrier contributing to premature cervical remodeling. Previous studies have also found that BCL2 is a direct target of miR-143 resulting in apoptosis in the cervical cancer cell line, HeLa, among others^41, 42^. Very little is known about the regulation and maintenance of the cervical epithelial barrier, however, in the gut, normal epithelial cell death occurs frequently in order to make room for new cells ensuring that the strongest epithelial cells are available to uphold the barrier. In disease states such as irritable bowel disease, significant pathological apoptosis is present^43, 44^. While the exact role of epithelial cell death on barrier integrity remains unclear in both the gut and the cervix, the consequences of this are hypothesized to be 1) disruption of the barrier allowing bacteria to traverse into the stroma, 2) loss of the anti-microbial (and other) protective factors released by epithelial cells and 3) epithelial cell death itself leads to increased inflammation and inflammatory cytokines creating a negative cycle of epithelial barrier breakdown.

Secondary to apoptosis, we also investigated the effects of miR-143 and miR-145 on cell proliferation and the cell cycle as a potential mechanism contributing to cervical epithelial barrier breakdown. Ectocervical and endocervical cells transfected with miR-143 and miR-145 showed cell cycle arrest at either G0/G1 or S phase. Additionally, direct gene targets, CDK1 and CCND2 were repressed in the presence of miR-143 and miR-145. CDK1 is a cyclin dependent kinase known to play an integral role in cell cycle regulation especially in the progression from G2 to M-phase. CCND2, also known as the G1/S-specific cyclin D2, is responsible for regulating the G1 to S-phase transition through the cell cycle. Nothing has been reported in the current literature pertaining to CDK1 and CCND2 in cervical or gut epithelial barrier integrity, however, it has been well established that epithelial cell proliferation is necessary in order to maintain a strong and intact epithelial barrier. Therefore, it is easy to hypothesize that increased expression of miR-143 and miR-145 in the cervicovaginal space could significantly decrease epithelial cell proliferation contributing to the breakdown of the cervical epithelial barrier.

Due to the known effects of miR-143 and miR-145 on cell growth, proliferation and apoptosis, these miRNAs have been highly studied in the cancer field. Interestingly, in agreement with our results, miR-143 and miR-145 have been shown to be tumor suppressors/oncogenic factors in human cancers of epithelial origin including cervical, colon, gastric, breast and pancreatic carcinomas due to their effects on inhibiting cell growth and proliferation and activating apoptosis^45–47^. These findings have led to several research studies investigating the therapeutic potential for both miR-143 and miR-145 which have shown promise in their ability to stop tumor growth^48, 49^. While overexpressing miR-143 and miR-145 has positive effects as a cancer therapy, in preterm birth, miR-143 and miR-145 would need to be inhibited in order to prevent premature breakdown of the cervical epithelial barrier and cervical remodeling. Nonetheless, these studies provide proof of principle that miR-143 and miR-145 could be potential biomarkers or therapy targets when administered directly into the cervical vaginal space.

When taken together, the results from this study show that increased expression of miR-143 and miR-145 in ectocervical and endocervical cells cause a breakdown in the epithelial barrier due to alterations in adherens junction proteins and a significant reduction in cell number due to cervical cell apoptosis and cell cycle arrest. As we know from our previous work that miR-143 and miR-145 expression is upregulated in cervical epithelial cells collected from women destined to have a preterm birth, we can conclude that it is biologically plausible that miR-143 and miR-145 contribute significantly to premature cervical remodeling due to a disruption in epithelial barrier integrity that ultimately leads to early delivery. This research study has identified multiple molecular pathways that are altered in the presence of increased miR-143 and miR-145. While miR-143 and miR-145 undoubtedly target hundreds of downstream genes and, consequently, have the widespread ability to effect many gene networks, it is clear that these miRNAs target pathways that contribute directly to cervical epithelial barrier integrity. Understanding the molecular pathways regulating cervical remodeling are critical to devising future therapies aimed at reducing the incidence of preterm birth.

## Methods

### Cell Culture

Transformed ectocervical (Ect/E6E7, AATC# CRL-2614) (Ecto) and endocervical (End1/E6E7, AATC# CRL-2615) (Endo) cell lines (American Type Culture Collection, Bethesda, MD) were cultured in Kerotinocyte-Serum Free Media (K-SFM) supplemented with 0.1 ng/ml epidermal growth factor and 50 ug/ml bovine pituitary extract (ScienCell Laboratories, Carlsbad, CA), 100 U/mL penicillin, and 100 μg/mL of streptomycin at 37 °C in a 5% CO_2_ humidified incubator. Human embryonic kidney 293T (HEK293T) cells (American Type Culture Collection) used in the 3′UTR reporter assays were maintained in Dulbecco’s Modified Eagle Medium (Gibco, Thermo-Fisher Scientific, Waltham, MA) supplemented with 10% charcoal-stripped (steroid-free) fetal bovine serum (FBS) and 100 U/mL penicillin, and 100 μg/mL of streptomycin at 37 °C in a 5% CO_2_ humidified incubator.

### Ectocervical and Endocervical Cell Transfection

Ectocervical cells were plated at 1.5 × 10^5^ cells/well and endocervical cells were plated at 2 × 10^5^ cells/well in 6-well plates in antibiotic-free K-SFM media. After 24 hrs, the cells were transfected with miRNA mimics (20 uM, final concentration of 40 nM). Hsa-miR-143–3p (miR-143), hsa-miR-145-5p (miR-145) and miR-negative (miR-neg, non-targeting control) miRNA mimics were purchased from Ambion (Applied Biosystems, Thermo-Fisher Scientific). Lipofectamine RNAiMAX (Invitrogen, Thermo-Fisher Scientific) was used for the transfection of the miRNA mimics according to the manufacturers’ protocol. Cells were transfected for 48 to 72 hours and maintained under normal growth conditions. Efficiency of miR-143 and miR-145 transfection was verified by QPCR at the 72 hour time point (see Supplementary Fig. S1). Transfected cells were then used for cell permeability assays, immunohistochemistry, apoptosis or cell proliferation assays by flow cytometry or QPCR measurement of miRNAs or target genes.

### Epithelial Cell Permeability Experiments

Endocervical and ectocervical cell permeability was determined using an *In Vitro* Vascular Permeability Assay (Millipore, Bedford, MA). Briefly, miR-neg, miR-143 and miR-145 transfected endocervical and ectocervical cells were plated at 1.0 × 10^6^ cells/ml respectively into 24 well hanging cell culture inserts which contain 1 µm pores with a transparent polyethylene terephthlate (PET) membrane pre-coated with collagen. After 24, 48 and 72 hours of growth, an insert membrane from one representative well was stained and analyzed by brightfield imaging to assess monolayer integrity. At 72 hours, when a monolayer was completely formed, the media was removed and phenol red free K-SFM media (ScienCell Laboratories, Carlsbad, CA) containing FITC-Dextran was added to the top of the insert. The movement of FITC-Dextran from the top insert to the bottom was measured after two hours by a fluorescent plate reader at 485 nm and 535 nm, excitation and emission, respectively.

### mRNA and miRNA Isolation from Ectocervical and Endocervical Cells

Following miR-neg, miR-143 and miR-145 transfection, ectocervical and endocervical cells were washed in sterile PBS, collected in TRIzol (Invitrogen, Thermo-Fisher Scientific) and underwent phenol-chloroform extraction. The resulting aqueous phase was further column purified with the miRNeasy kit (Qiagen, Hilden, Germany) according to the manufacturer’s protocol for total RNA isolation including small RNAs. RNA concentration was determined via a NanoDrop 2000 Spectrophotometer (Nanodrop^TM^ Rockland, DE) prior to the generation of cDNA.

### cDNA generation and qPCR

cDNA was generated from 1 µg of isolated miRNA/mRNA from ectocervical and endocervical cells (both *in vitro* cell lines and RNA PAP samples) using the miScript Reverse Transcription II kit (miRNA) (Qiagen) or high capacity cDNA reverse transcription kit (mRNA) (Applied Biosystems, Thermo-Fisher Scientific) for SYBR Green or TaqMan primers, respectively. qPCR was performed on the 7900HT Real-Time PCR System (Applied Biosystems) using the miScript SYBR Green PCR kit (Qiagen) or TaqMan Universal PCR Master Mix (Applied Biosystems) according to the manufacturers’ protocols. The ΔΔCT (SYBR Green PCR) or standard curve (TaqMan PCR) method was used for relative expression quantification using the RQ manager software v2.4 (Applied Biosystems). For SYBR Green PCR, the endogenous reference gene RNU6B was used for miRNA quantification. All miRNA primer sets were purchased from Qiagen: miR-143 (MS00003514), miR-145 (MS00003528) and RNU6B (MS0001400). For TaqMan PCR, the relative abundance of the target of interest was divided by the relative abundance of 18S in each sample to generate a standardized abundance for the target transcript of interest. All mRNA primers were purchased from Applied Biosystems: JAM-A (F11R), FSCN1, BCL2, BIRC5, CDK1, CCND2 and 18S.

### Western Blot

Ectocervical and endocervical cells were plated at 2 × 105 cells/well in 6-well plates. The cells were transfected with miR-negative control (miR-neg), miR-143 or miR-145 as described above for 72 hours. The cells were rinsed with ice cold PBS at the time of harvesting and whole cell protein lysates were extracted with ice-cold RIPA buffer containing protease inhibitors (Complete Mini Tablets, Roche Diagnostics, Indianapolis, IN). Protein concentrations were measured using the Pierce Bicinchoninic Acid (BCA) assay (ThermoScientific, Rockford, IL) according to the manufacturer’s protocol. 25 ug of each protein lysate was resolved via SDS-PAGE and transferred to PVDF membranes. The membranes were blocked for 1 hour at room temperature with blocking buffer (5% milk, 0.5% Tween20, 1X Tris-buffered saline (BioRad, Hercules, California)) and then probed with rabbit monoclonal primary antibodies (see Table 3) in blocking buffer at 4 °C overnight. Membranes were rinsed three times each with wash buffer (0.5% Tween20, 1X Tris-buffered saline) and then probed with anti-rabbit IgG HPR-linked secondary antibody (GE Healthcare, Piscataway, NJ, 1:1000) in blocking buffer for one hour at room temperature. Membranes were rinsed three times in wash buffer. SuperSignal West Pico Chemiluminescent Substrate (ThermoScientific) was added to the membranes according to the manufacturer’s instructions. Membranes were imaged using the ChemiDocMP Imaging System (BioRad). All membranes were stripped and reprobed for the loading control Beta-Actin (secondary antibody: Goat anti-mouse IgG, Jackson Laboratories, 1:10,000) using BlotFresh Western Blot Stripping Reagent (SignaGen Laboratories, Gaithersburg, MD) according to the manufacture’s protocol.

**Table 3:** List of primary antibodies used for western blots.

Protein	Antibody	Catalog Number	Company	Antibody Dilution	Protein Size
JAM-A	Anti-Junctional Adhesion Molecule 1 antibody [EP1042Y]	ab52647	Abcam	1:4000	33 KDa
FSCN1	Anti-Fascin antibody [EP5902]	ab126772	Abcam	1:25,000	54 KDa
BCL2	Anti-BCL2 [EPR17509]	ab182858	Abcam	1:600	26 KDa
BIRC5	Anti-Survivin antibody [EP2880Y]	ab76424	Abcam	1:2000	16 KDa
CDK1	Anti-CDK1 antibody [EPR165]	ab133327	Abcam	1:2500	34 KDa
CCND2	Anti-Cyclin D2 antibody [EPR19659]	ab207604	Abcam	1:1000	33 KDa
Beta-Actin	Beta-Actin Loading Control Antibody	MA5–15739	ThermoFisher Scientific	1:1000	42 KDa

### Immunocytochemistry

Glass chamber slides were coated with 0.1% gelatin (Sigma, St. Louis, MO) for two hours prior to plating the ectocervical and endocervical cells. The cells were plated at 1.5 × 10^5^ cells/ml onto the gelatin-coated slides. Ectocervical and endocervical cells were transfected with miR-neg, miR-143 and miR-145 for 48 hours. The cells were fixed with 10% buffered formalin for 30 minutes, washed with cold PBS and permeabilized using 0.25% triton-X for 10 minutes. The slides were blocked with 5% donkey serum for 30 minutes and incubated with primary antibody, human JAM-A polyclonal anti-goat (1:50, R&D systems, Minneapolis, MN) overnight at 4 °C. The next day, the slides were washed three times with cold PBS and incubated with secondary antibody, AlexaFluor 568 Donkey anti-goat (1:750, Life Technologies) for one hour at room temperature. After washing, the slides were stained with DAPI (1:500, Molecular Probes, Eugene, OR) for one hour. The slides were washed again, dehydrated and mounted with Krystalon (Harleco, Darmstadt, Germany). To validate the staining procedure, ectocervical and endocervical cells were stained for JAM-A in duplicate slides with each slide containing a section incubated with and without (negative control) the primary antibody. Stained cells were photographed on a Zeiss LSM 710 confocal system set up on an AxioObserver inverted microscope.

### Apoptosis Assay

Apoptosis in transfected ectocervical and endocervical cells was measured by flow cytometry using a FITC Annexin V/PI apoptosis detection kit (Invitrogen, Thermo-fisher Scientific) according to the manufacture’s protocol. Ectocervical and endocervical cells transfected for 0, 72 and 144 hours were washed in cold sterile PBS, pelleted, counted (recorded for cell number determinations) and resuspended in 1X annexin-binding buffer at a concentration of 1 × 10^5^/100 ul. The cells were incubated for 15 minutes with FITC annexin V and propidium iodide (PI, 100 ug/ml) and then further diluted with 400 ul 1X annexin-binding buffer. The stained cells were then analyzed by a flow cytometer (Accuri C6, BD Biosciences, San Jose, CA) using a fluorescence emission at 530 nm (FL1) and >575 nm (FL3). Single color stains for FITC annexin and PI and unstained cells were included in all experiments as positive and negative controls.

### 3′UTR Luciferase Reporter Assay

HEK293T cells were plated at 2.0 × 10^5^ cells/well in 12 well plates. After 24 hours, miTarget miRNA 3′UTR Target clones specific for BCL2, BIRC5, JAM-A, FSCN1, CDK1 or CCND2 inserted into a pEZX-MT05 vector (Genecopoeia, Rockville, MD) were transfected into the HEK293T cells using 3 ul of Lipofectamine 3000 (Invitrogen, Thermo-Fisher Scientific). The pEZX-MT05 vector contains a Gaussia luciferase (GLuc) reporter gene driven by an SV40 promoter and a secreted Alkaline Phosphatase (SEAP) reporter driven by a CMV promoter which serves as the internal control for transfection efficiency. Twenty four hours after transfection with the reporter plasmid, miR-neg, miR-143 or miR-145 were transfected into the cells using Lipofectamine RNAiMAX as described above. Twenty four hours later, media was collected for GLuc and SEAP activities using the Secrete-Pair Gaussia Luciferase Assay Kit (Genecopoeia). GLuc and SEAP activity was measured by a luminescent plate reader. EF1A-PG04 media (provided by Genecopoeia) was included in all GLuc measurements as a positive control for the assay. The results are expressed as a ratio of GLuc to SEAP and normalized to cells transfected with the target gene plasmid alone.

### Cell Cycle Assay

Alterations in cell cycle progression was assessed after 72 hours of miR-neg, miR-143, and miR-145 transfection in ectocervical and endocervical cells by flow cytometry. After transfection, cells were washed in sterile PBS, pelleted, counted and resuspended in 70% ethanol at a concentration of 1 × 10^6^ cells/ml for 30 minutes at 4 °C to fix the cells. The cells were washed and centrifuged twice and resuspended in 500 ul of FxCycle Propidium Iodide/RNase staining solution (Molecular Probes, Life Technologies) and incubated for 30 minutes. The samples were analyzed by a flow cytometer (Accuri C6) using the FL3 channel. For cell cycle analyses the cells were first gated on the FSC/SSC plot. Doublets and sub-G0 events were gated out on the PI-H/PI-A plot (where H and A stand for the height of the pulse and the area of the pulse, respectively).

### Statistical Analysis

Statistical analyses were performed for all experiments with the GraphPad Prism Software (Version 4.0, San Diego, CA). For data that were normally distributed, one-way analysis of variance (ANOVA) or two-way analysis of variance (ANOVA) was used. If statistical significance was reached (p < 0.05), then pair-wise comparison with a Tukey (one-way ANOVA) or Bonferroni (two-way ANOVA) post hoc test was performed. If data were not normally distributed, then the non-parametric Mann-Whitney test (one-way ANOVA) was used.

## Electronic supplementary material

Supplemental info and data
