# Supplementary material for: miR-143 and miR-145 disrupt the cervical epithelial barrier through dysregulation of cell adhesion, apoptosis and proliferation
Source: Sci Rep. 2017 Jun 8;7:3020. doi: 10.1038/s41598-017-03217-7 (PMC5465080; doi:10.1038/s41598-017-03217-7)

miR-143 and miR-145 disrupt the cervical epithelial barrier through dysregulation of cell adhesion, apoptosis and proliferation

Authors: Lauren Anton[1*], Ann DeVine[1], Luz-Jeannette Sierra[1], Amy G. Brown[1], Michal A. Elovitz[1]

[1] Maternal and Child Health Research Program, Department of Obstetrics and Gynecology, Perelman School of Medicine at the University of Pennsylvania, Philadelphia, PA 19104, USA.

Supplementary Methods

ApoTox-Glo Triplex Assay: The mechanism of endocervical (n=3) and ectocervical (n=3) cell death was determined using the ApoTox-Glo triplex assay (Promega, Madison, WI). The ApoTox-Glo triplex assay is able to assess viability, cytotoxicity and caspase activation in a single sample. Ectocervical and endocervical cells were plated in 6-well plates at $2x10^5$ cells/well and transfected with miR-negative control, miR-143 and miR-145 for 0 and 72 hours as described in the materials and methods section. The cells were then plated into a black 96-well plate at $3x10^4$ cells/ml for 5 hours prior to starting the triplex assay. The triplex assay was performed as specified by the manufacturer's protocol. Viability and cytotoxicity were measured by a fluorescent plate reader at 400 excitation and 505 emission (viability) and 485 excitation and 520 emission (cytotoxicity). Apoptosis (caspase activation) was measured by luminescence. Ectocervical and endocervical cells treated with ionomycin (100uM) and staurosporine (10uM) acted as positive controls for cytotoxicity and apoptosis, respectively. Media alone and non-transfected cells acted as negative controls.

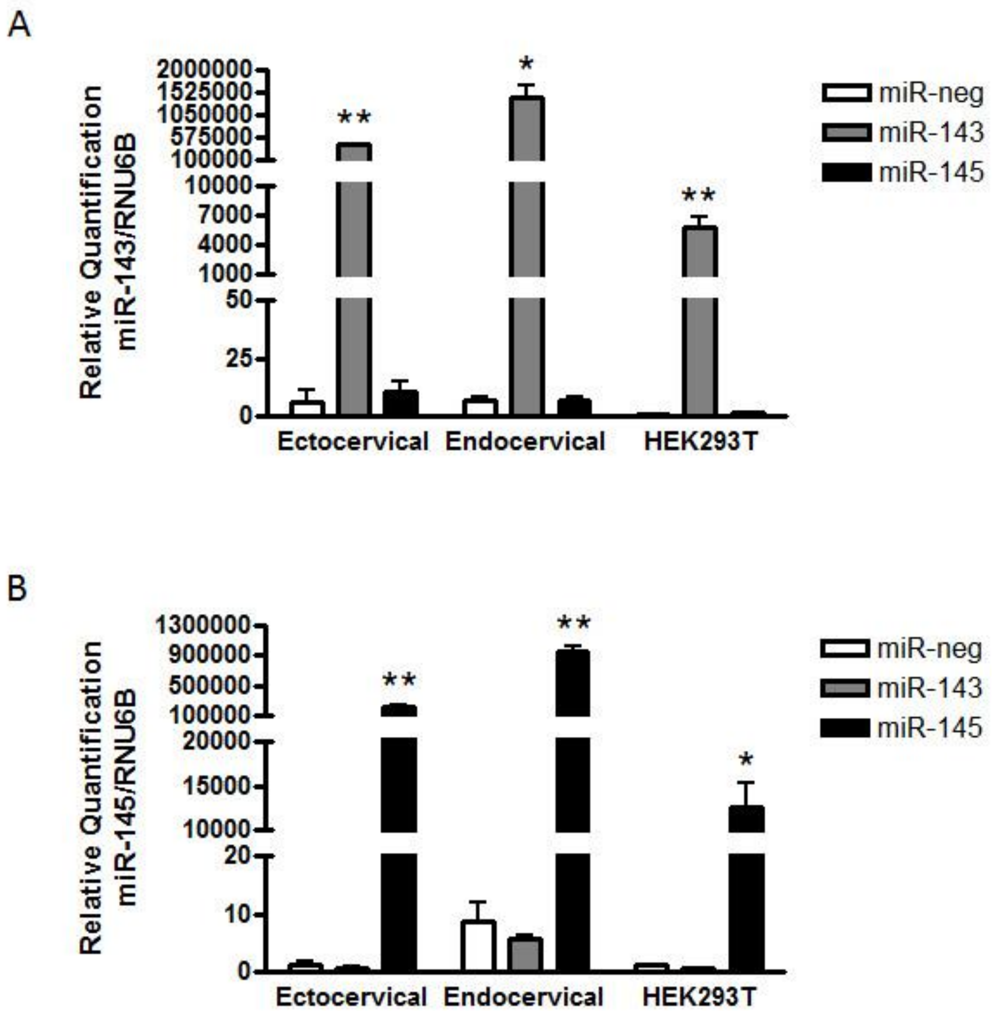

Supplementary Figure S1: miR-143 and miR-145 transfection efficiency of ectocervical, endocervical and HEK293T cells. Ectocervical (n=3), endocervical (n=3) and HEK293T (n=3) cells were transfected with miR-negative control (miR-neg), miR-143 and miR-145 mimics for 72 hours and expression of miR-143 and miR-145 were measured by QPCR. Ectocervical, endocervical and HEK293T cells were readily transfected to overexpress miR-143 (A) or miR-145 (B) with great specificity. Values are mean ± SEM. *p<0.01, **p<0.001

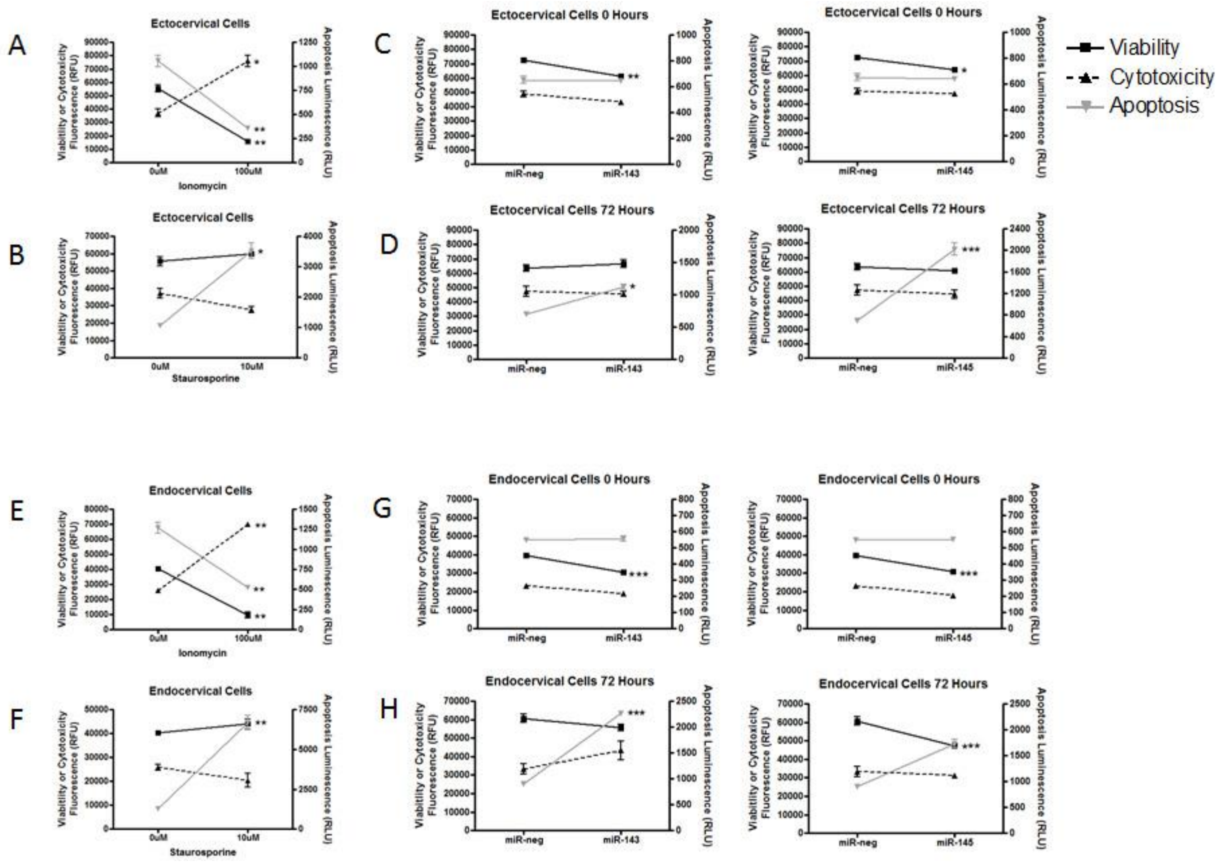

Supplementary Figure S2: miR-143 and miR-145 induce apoptosis of ectocervical and endocervical cells. Ectocervical and endocervical cells were transfected with miR-negative control (miR-neg), miR-143 and miR-145 mimics for 0 and 72 hours and the triplex assay was performed. Treatment with the positive controls, ionomycin (A, E) and staurosporine (B, F), resulted in cytotoxicity and apoptosis, respectively, in both ectocervical and endocervical cells. Ectocervical cells transfected for 72 hours with both miR-143 and miR-145 resulted in a significant increase in apoptosis when compared to cells transfected with miR-neg (D). Similarly, endocervical cells transfected with miR-143 and miR-145 showed significant increases in apoptosis compared to those transfected with miR-neg (H). Alterations in apoptosis were not seen at the 0 hour time point in either cell type (C, G). The results of this assay suggest that the effect of miR-143 and miR-145 on ectocervical and endocervical cell number is at least partially due to increased apoptosis. Values are mean ± SEM. $*p<0.05$, $**p<0.01$, $***p<0.001$

Supplementary Table S1: Predicted miR-143 and miR-145 target genes with functions related to cell adhesion, apoptosis and proliferation

	Ectocervical Cells				Endocervical Cells				
	miR-143		miR-145		miR-143		miR-145		
Gene	Fold Change	P value	Fold Change	P value	Fold Change	P value	Fold Change	P value	Gene Ontology Annotation
PRC1	-1.38	0.0472	-2.15	0.0018	-1.50	0.0379	-1.64	0.0096	microtubule cytoskeleton organization, cytokinesis, spindle formation
MAPK7	-1.72	0.0075	1.58	0.0031	-1.66	0.0242	1.27	0.0659	negative regulation of apoptotic process, cell cycle
PLK1	-1.42	0.0086	-2.51	0.0001	-1.28	0.0519	-1.29	0.0308	G2/M transition of mitotic cell cycle, mitotic cytokinesis, cell proliferation, negative regulation of apoptotic process, G2 DNA damage checkpoint
TOP2A	-1.71	0.0008	-3.35	<0.0001	-1.62	0.0045	-1.58	0.0031	sister chromatid segregation, DNA topoisomerase complex, mitotic DNA integrity checkpoint, apoptotic chromosome condensation
TRAF6	-1.07	0.4770	1.17	0.1039	-1.14	0.4551	-1.13	0.4437	negative regulation of apoptotic process, positive regulation of NF-kappaB transcription factor activity
SMAD3	1.40	0.0155	1.28	0.0146	1.08	0.7089	1.05	0.6292	cell cycle arrest, transforming growth factor beta receptor signaling pathway, cell-cell junction organization, negative regulation of apoptotic process, regulation of epithelial cell proliferation
CDK6	-1.02	0.8777	-1.35	0.0470	1.11	0.2883	-1.37	0.0232	G1/S transition of mitotic cell cycle, positive regulation of cell-matrix adhesion
ADAM17	1.39	0.0069	-1.19	0.0785	1.61	0.0153	-1.10	0.5564	positive regulation of cell proliferation, positive regulation of cell growth, cell-cell junction
ROCK1	-1.01	0.9291	-1.37	0.0288	-1.11	0.4983	-1.29	0.0973	negative regulation of angiogenesis, actin cytoskeleton organization, regulation of cell adhesion
TGFBR2	-1.27	0.0789	-1.87	0.0017	-1.01	0.8813	-1.25	0.0362	positive regulation of cell proliferation

Gray shading denotes genes with a statistically significant altered expression of $p<0.05$ after transfection with miR-143 or miR-145 (compared to miR-negative control)

Western Blot Full Length Gels

JAM-A, exposure 5 minutes

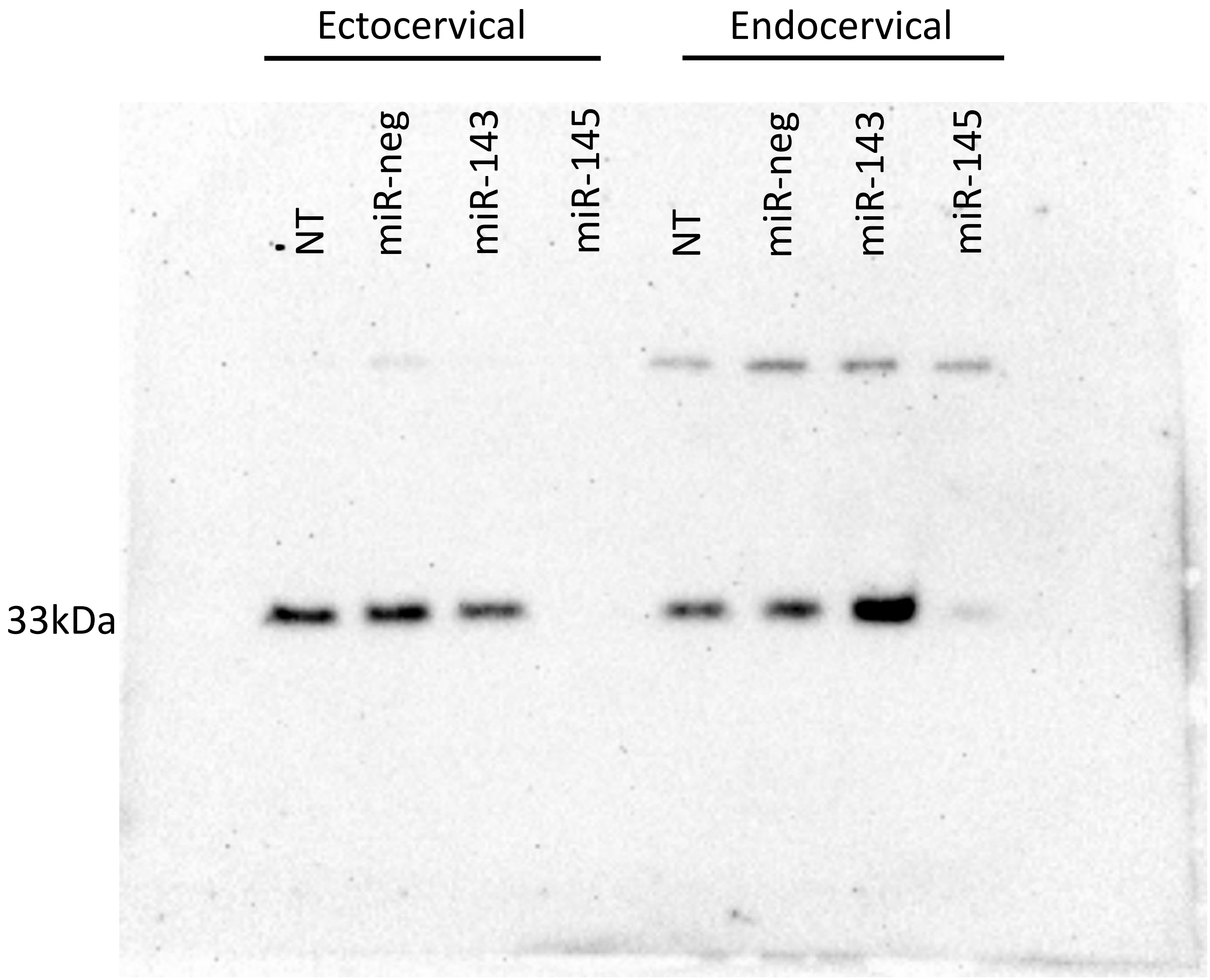

JAM-A Beta-Actin, exposure 2 minutes

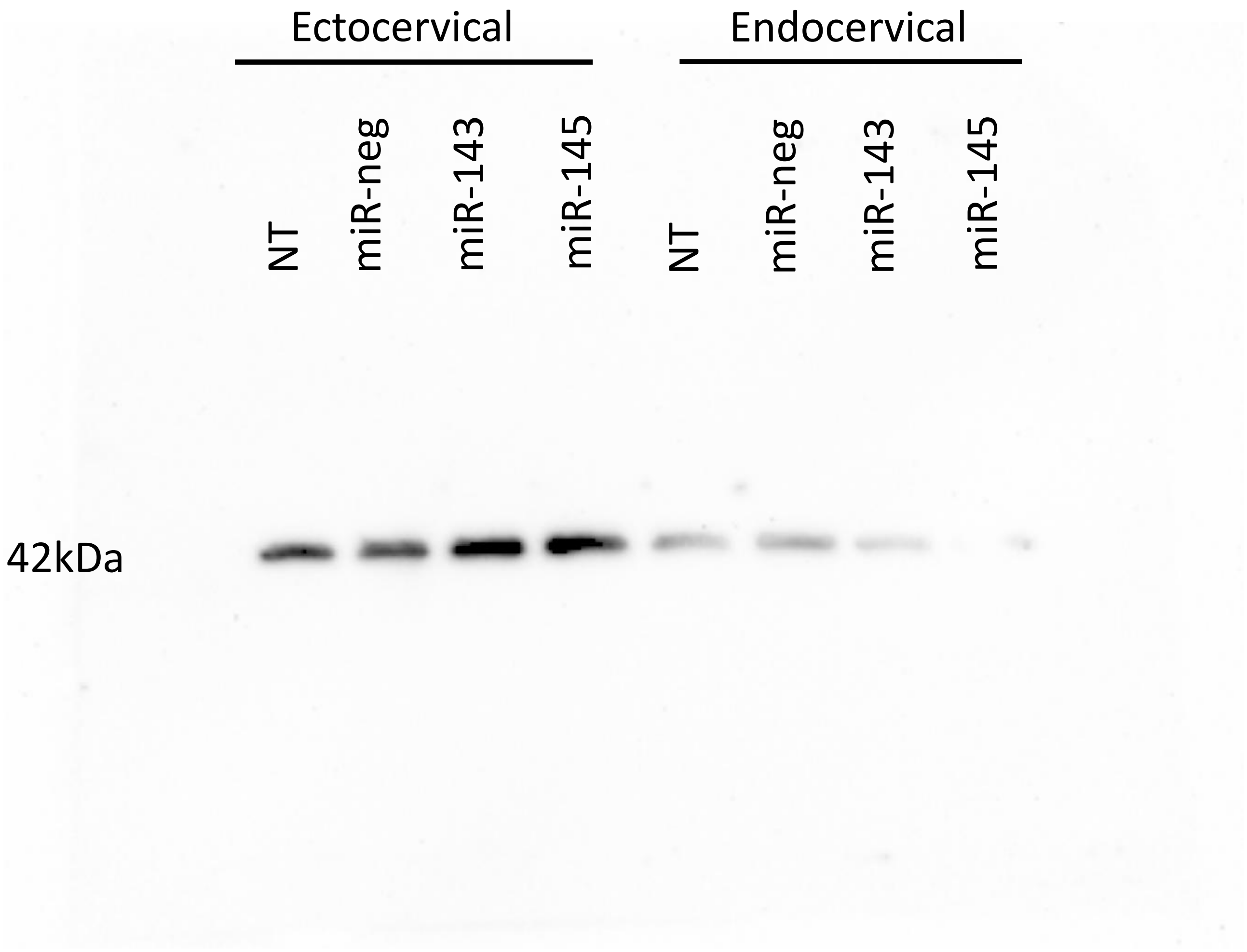

JAM-A Beta-Actin- Endo cells only, exposure 5 minutes

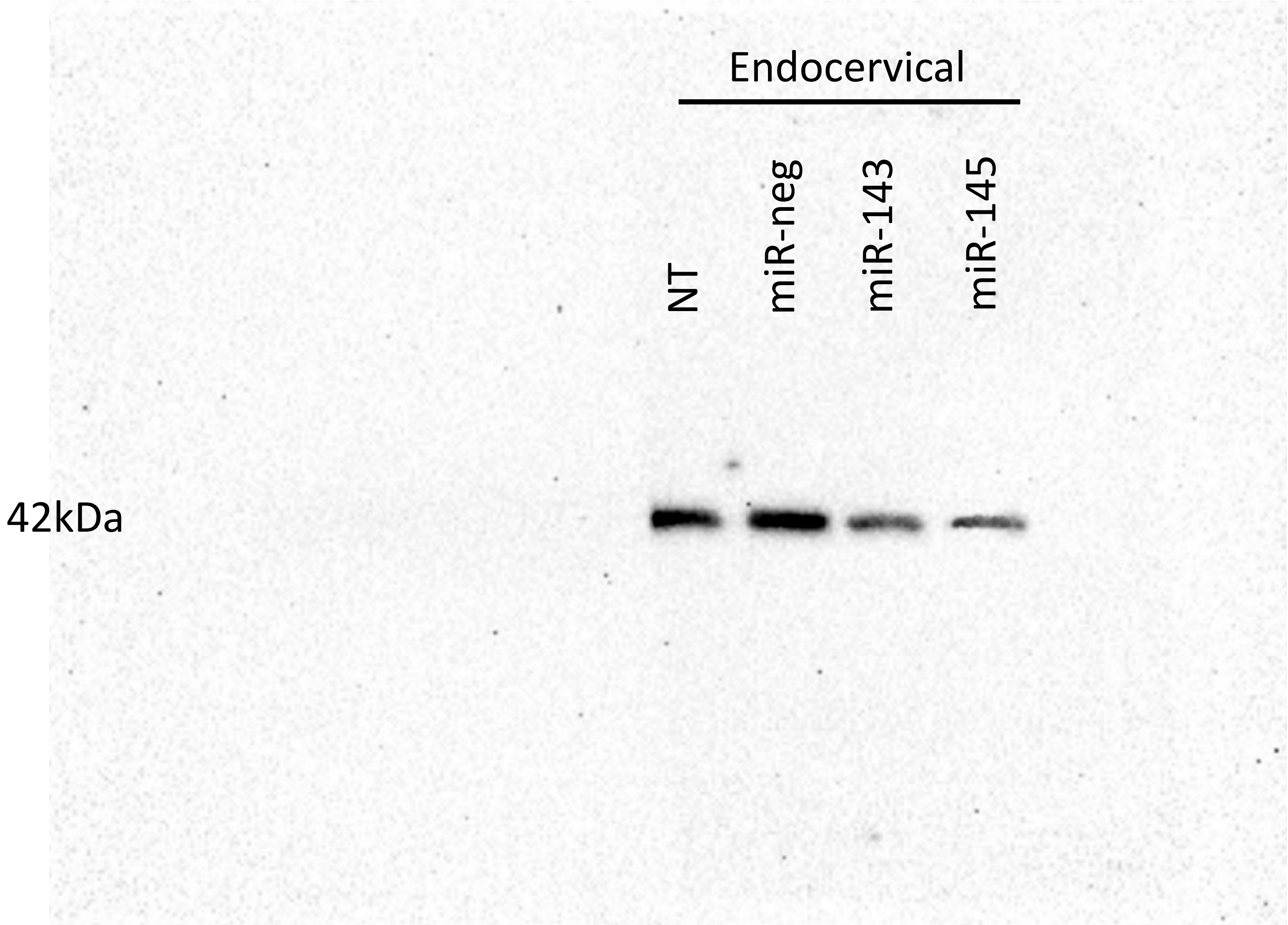

FSCN1, exposure 2 minutes

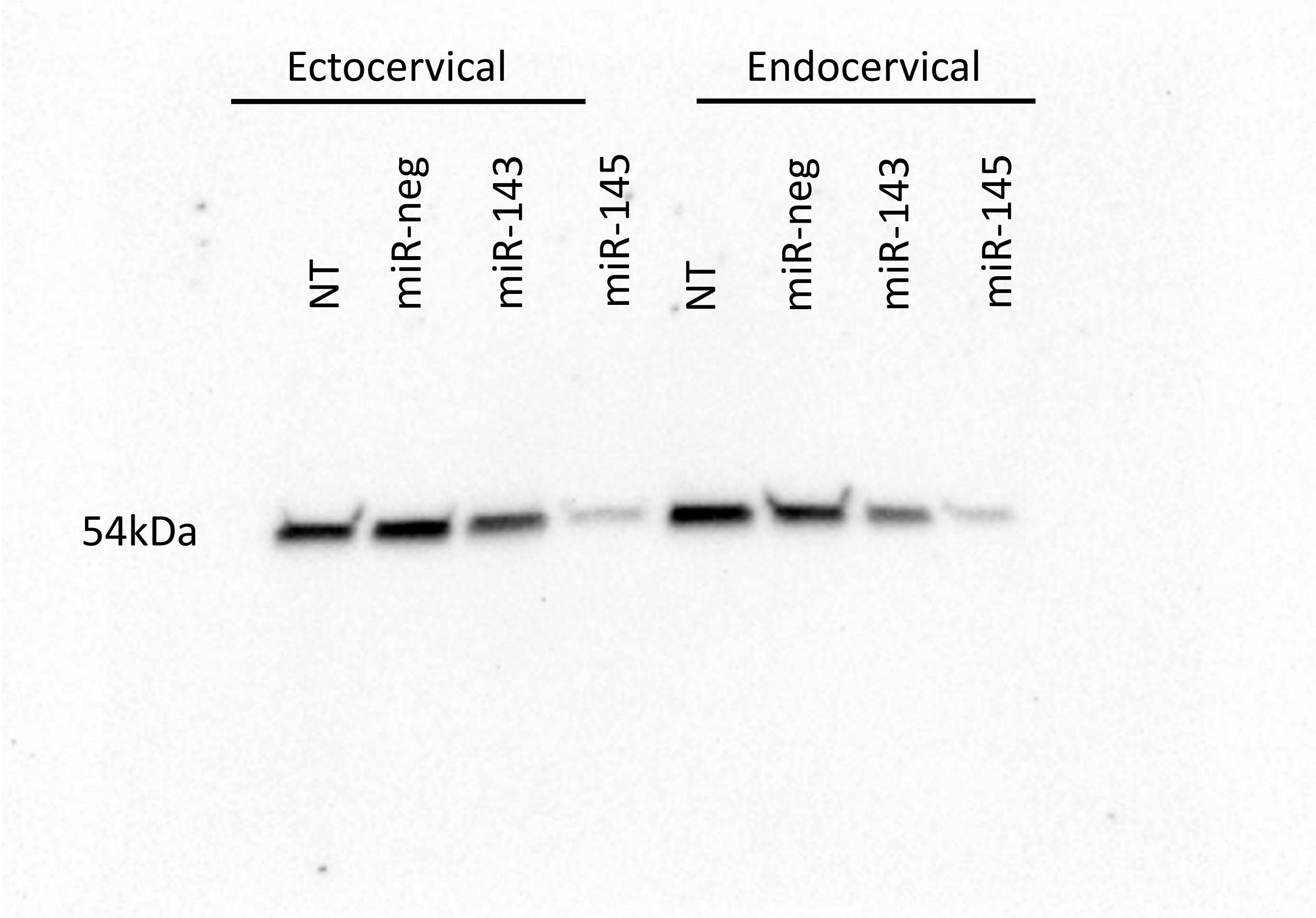

FSCN1 Beta-Actin, exposure 2 minutes

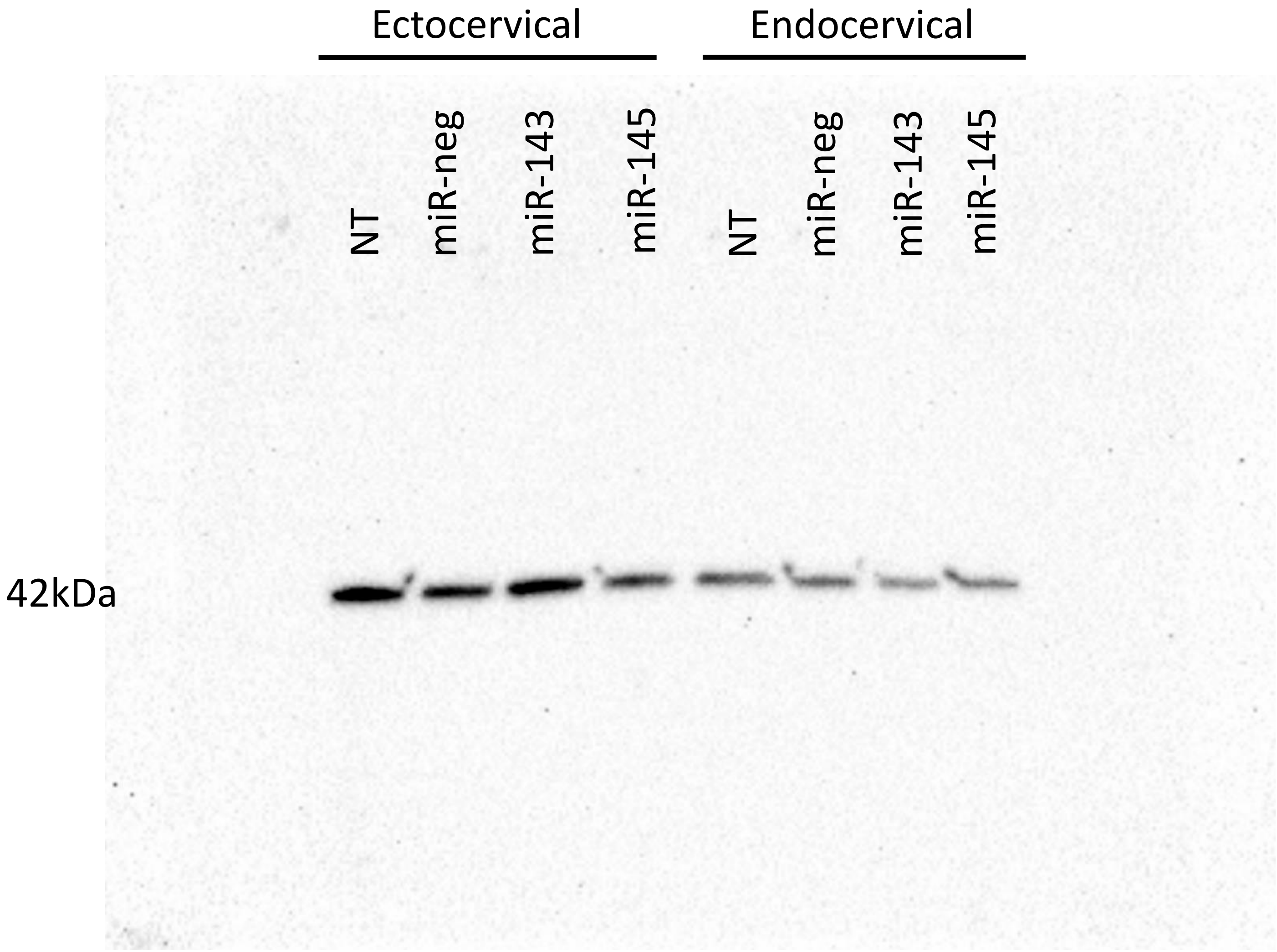

BCL2, exposure 10 minutes

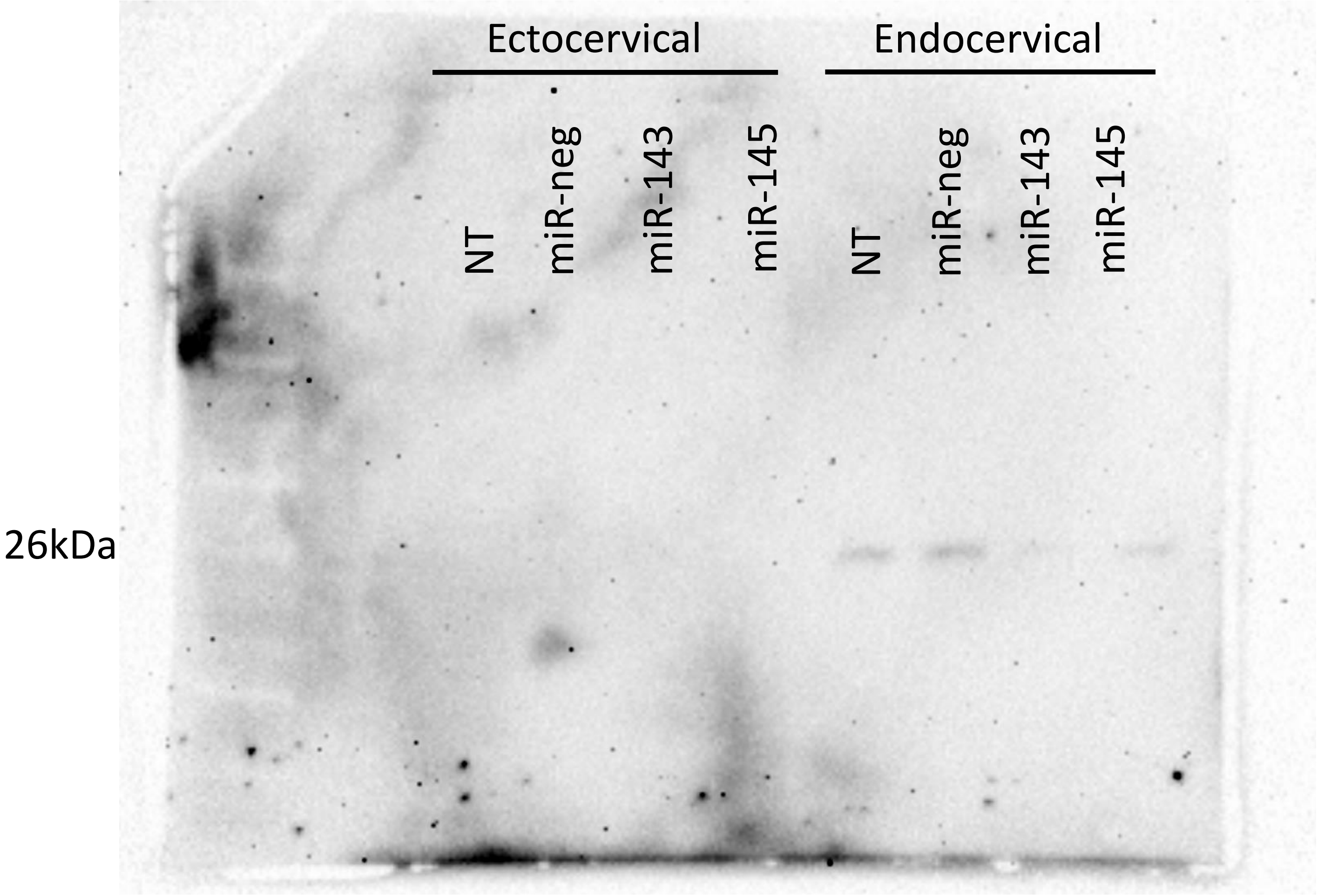

BCL2 Beta-Actin, exposure 3 minutes

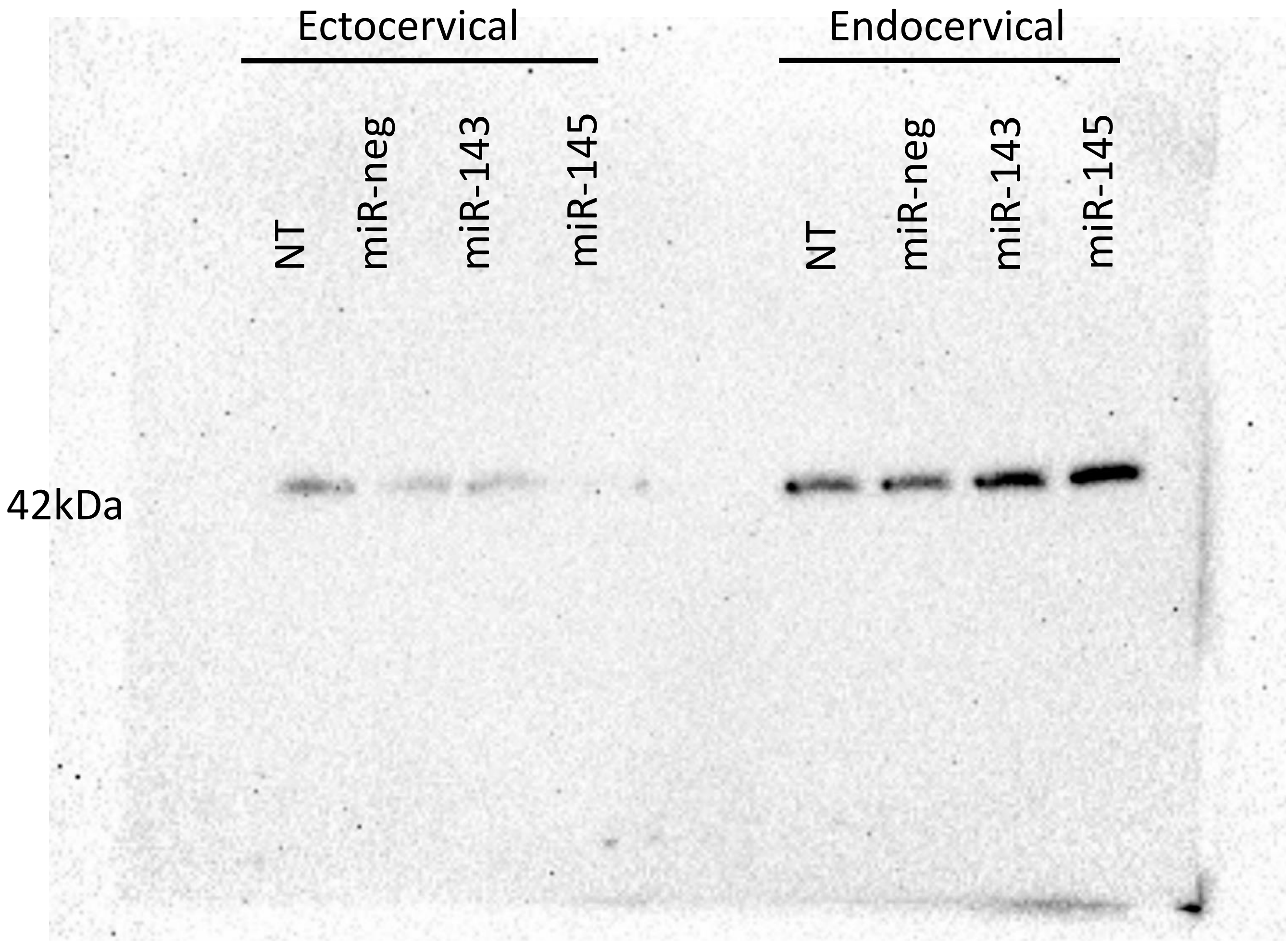

BCL2 Beta-Actin, ecto cells only, exposure 10 minutes

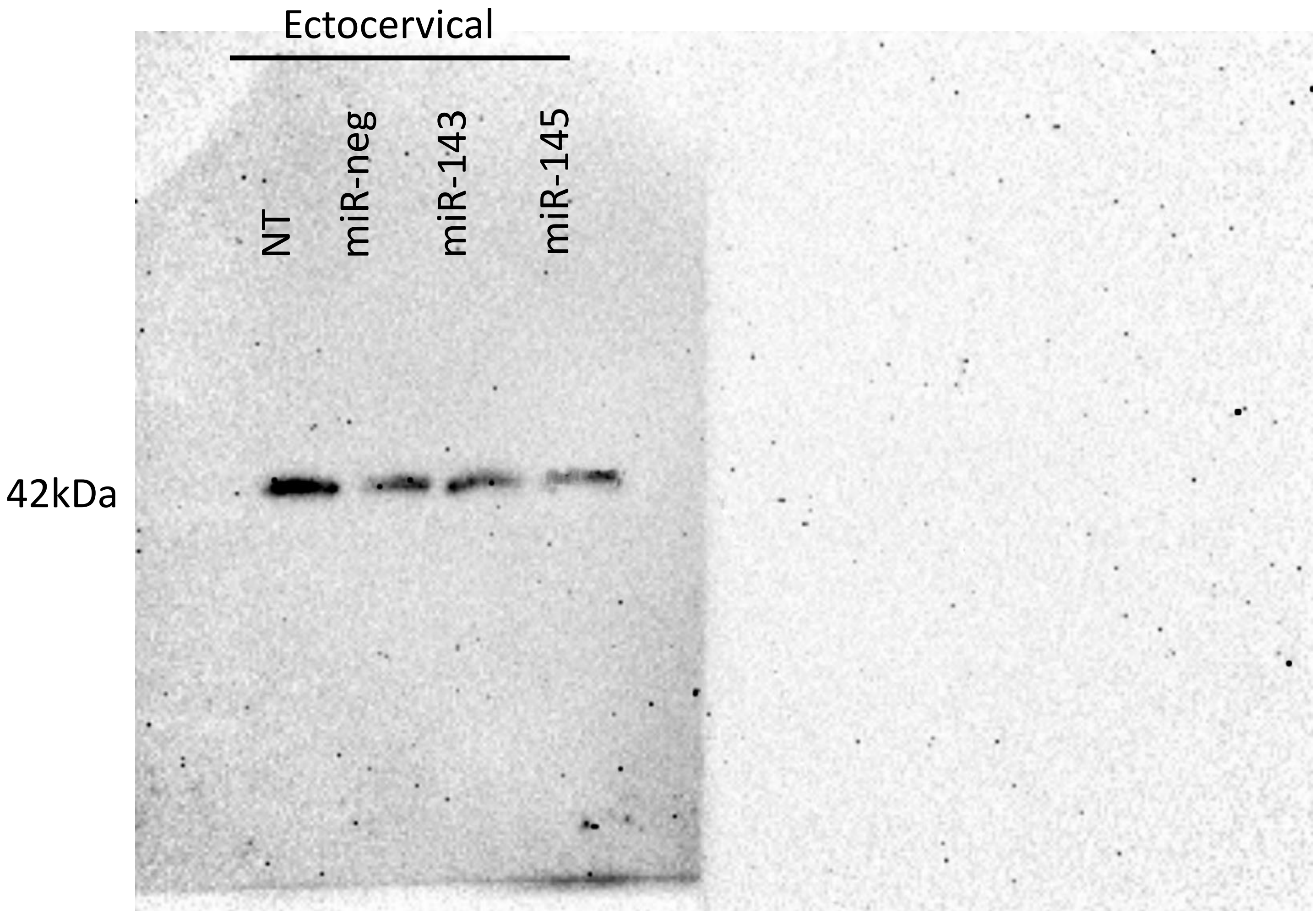

BIRC5, exposure 5 minutes

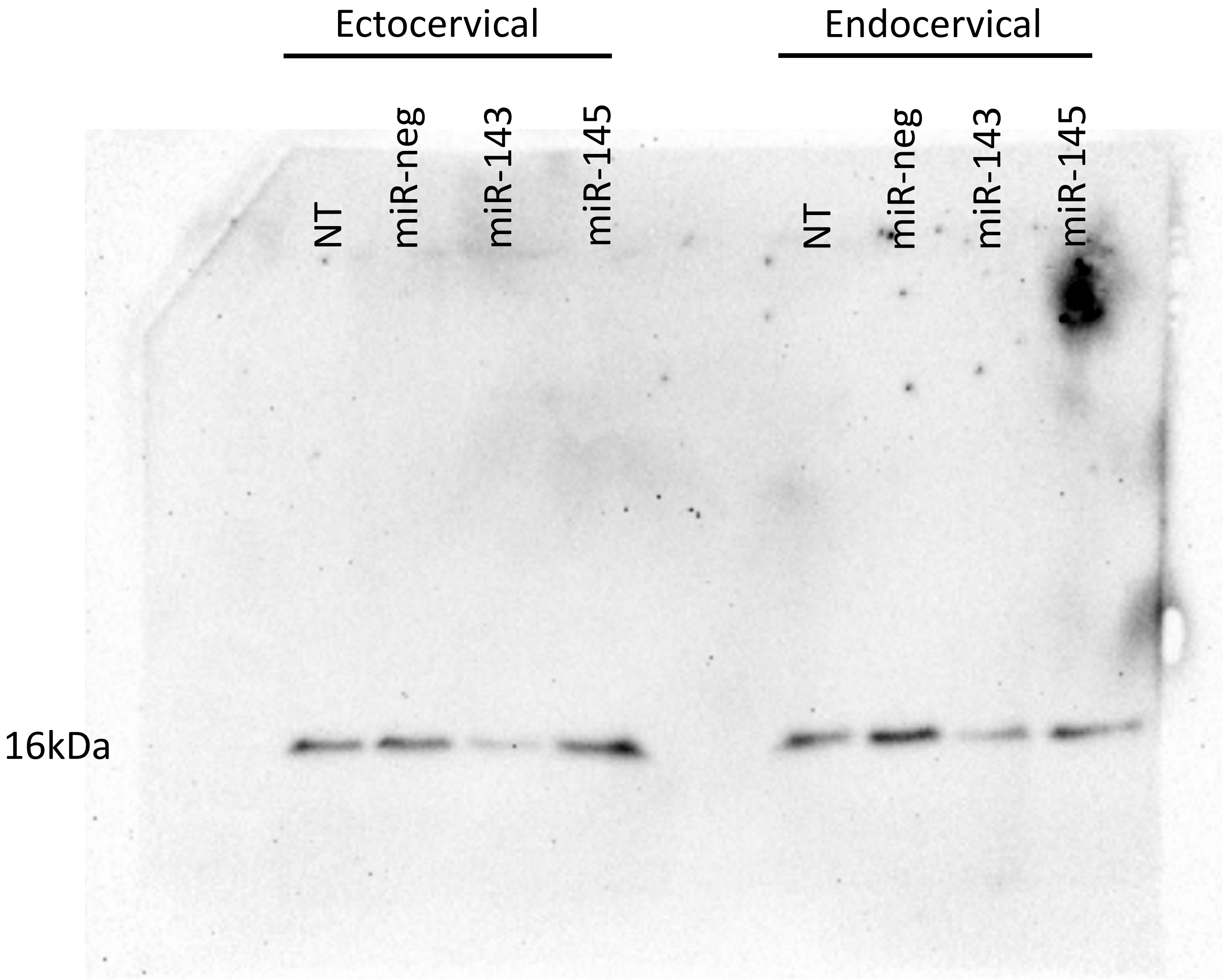

BIRC5 Beta-Actin, exposure 1 minute

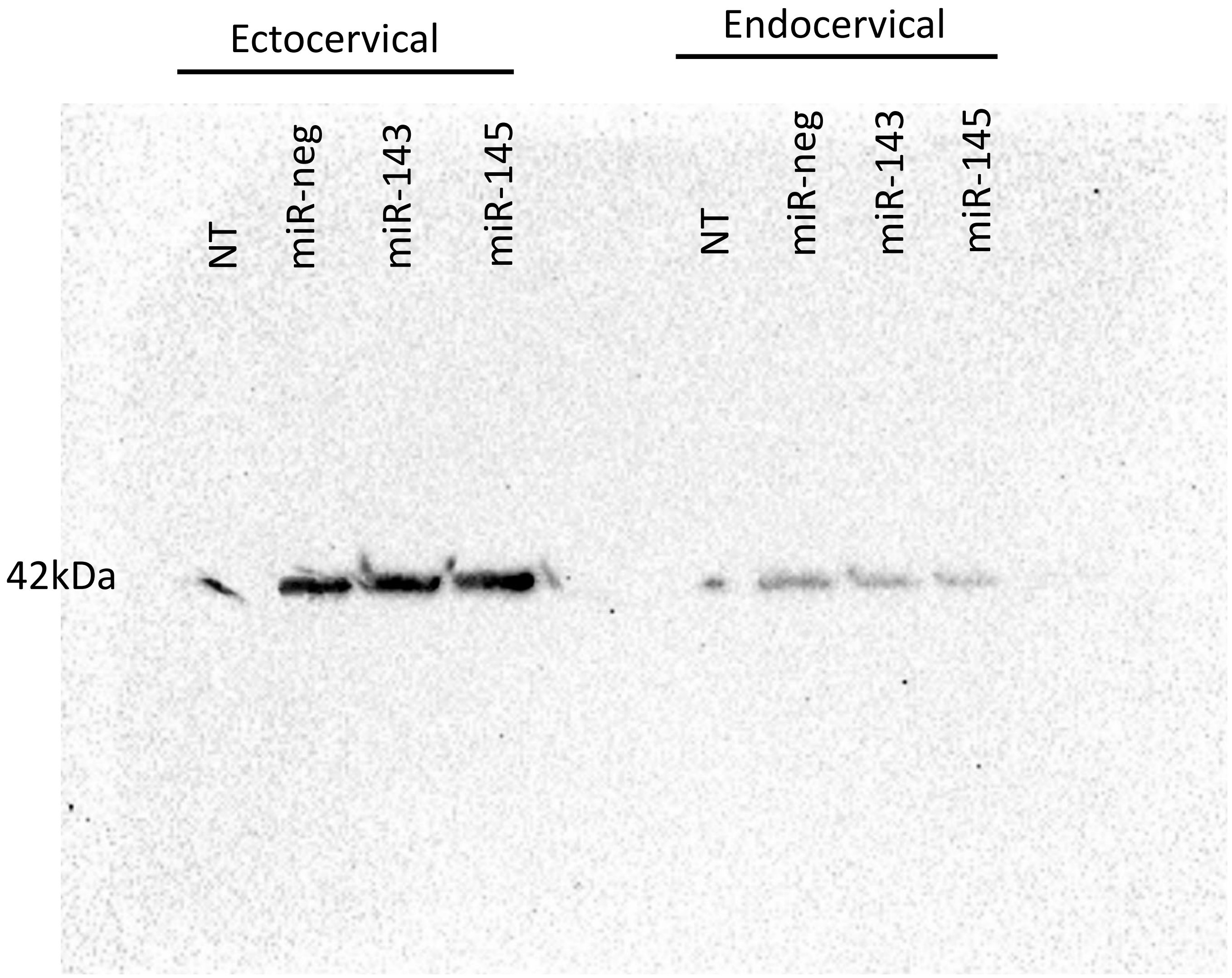

BIRC5 Beta-Actin, endo cells only, exposure 1 minute

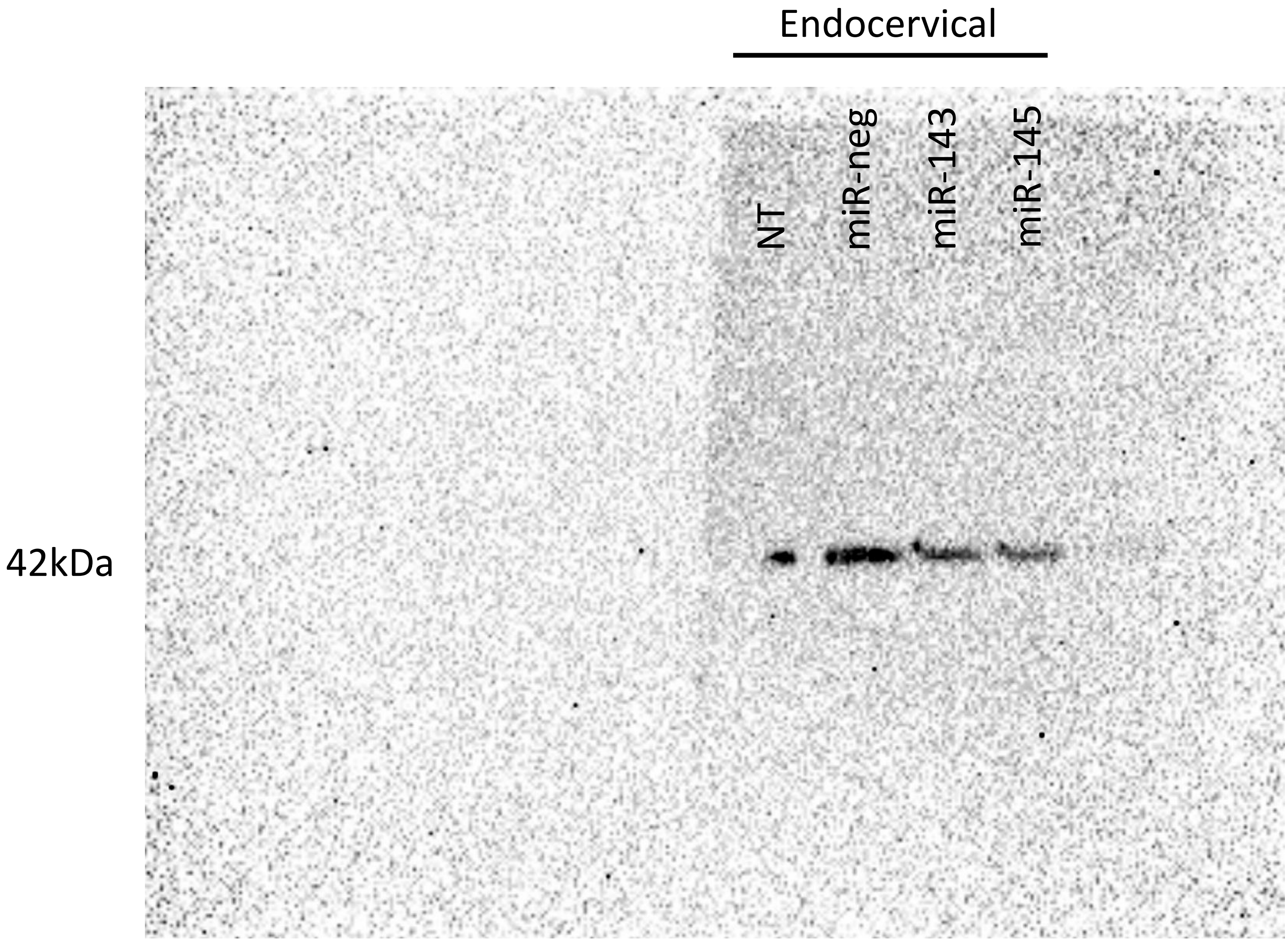

CDK1, exposure 4 minutes

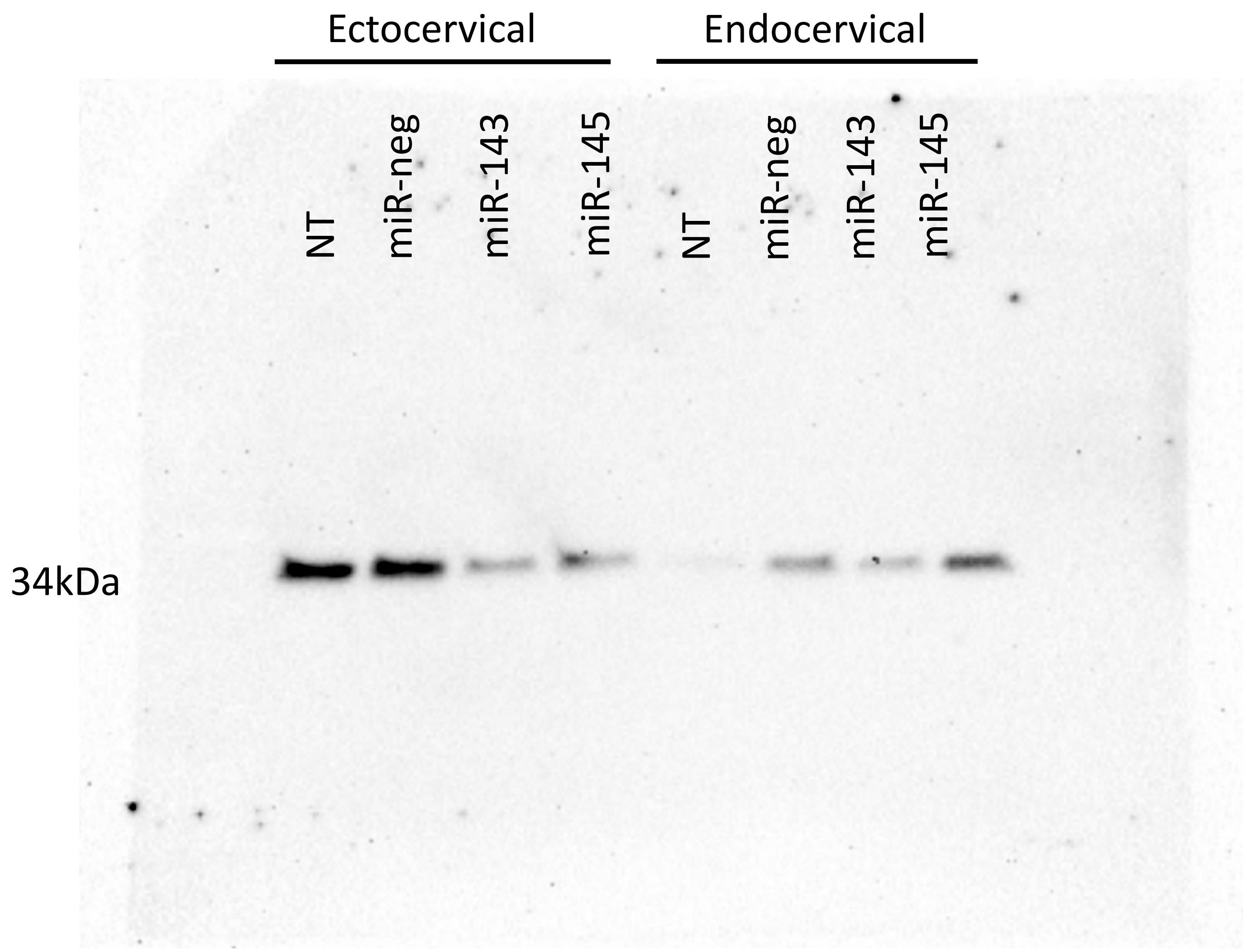

CDK1 Beta-Actin, exposure 1 minute

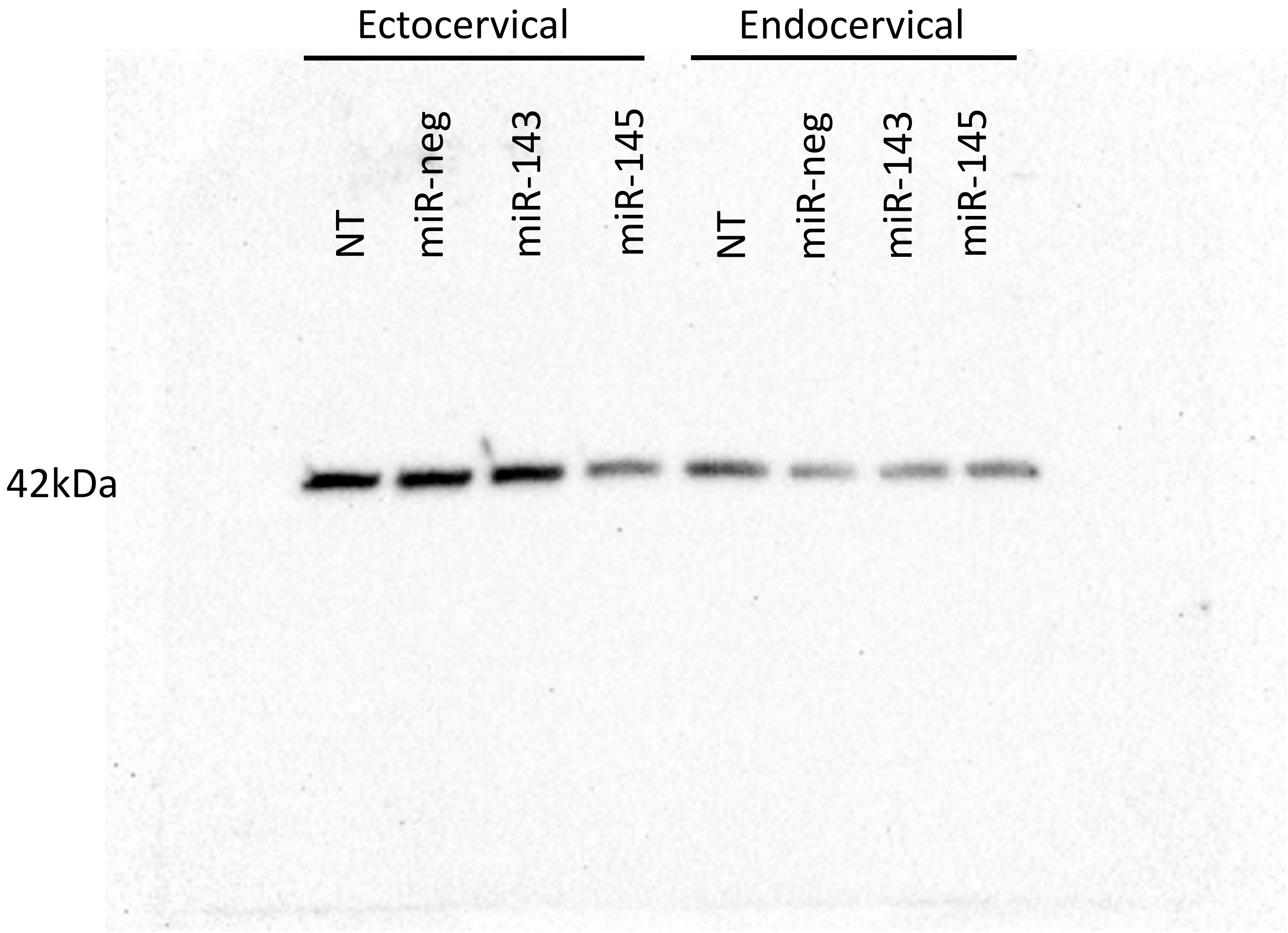

CCND2, exposure 5 minutes

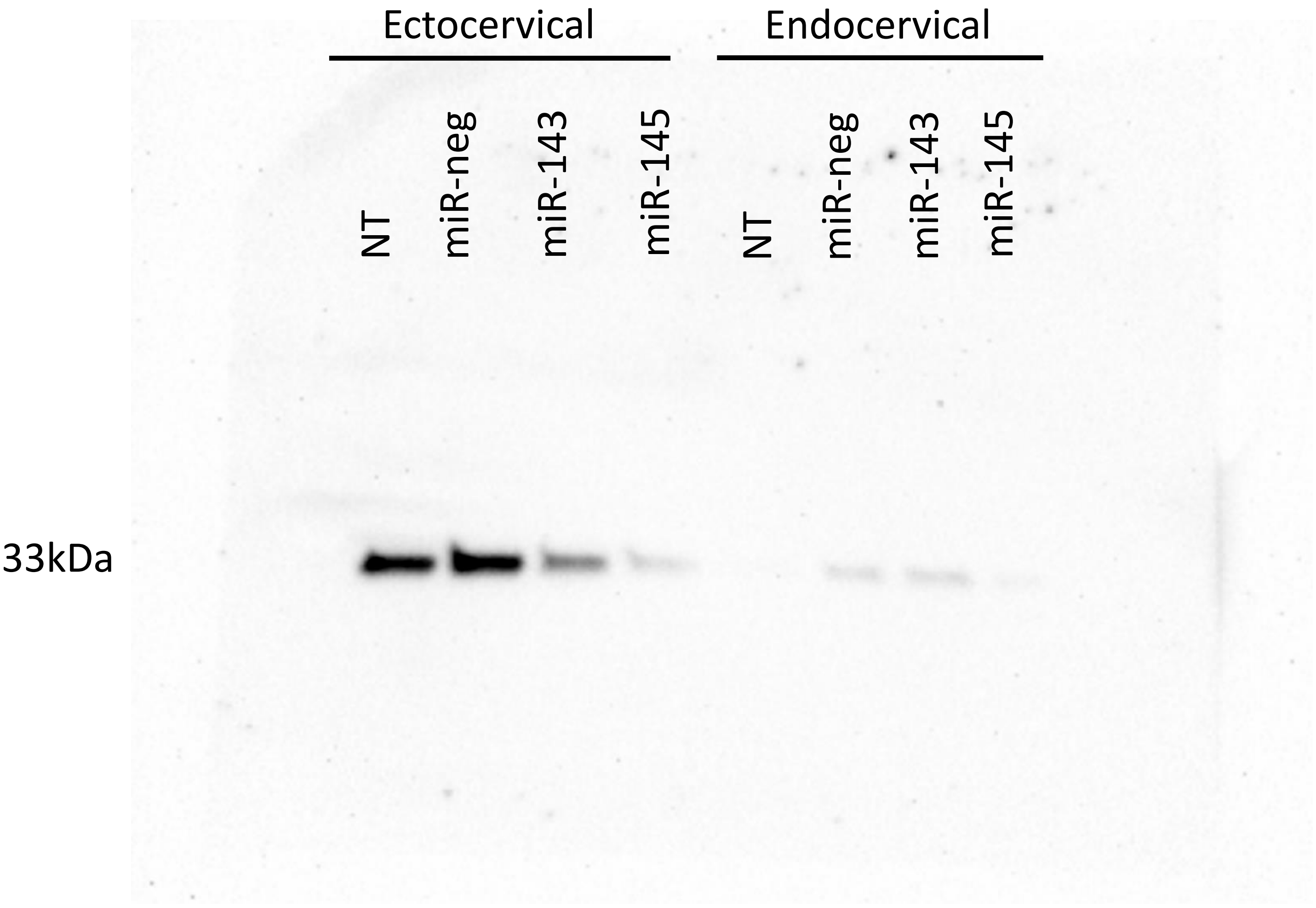

CCND2, exposure 1 minute

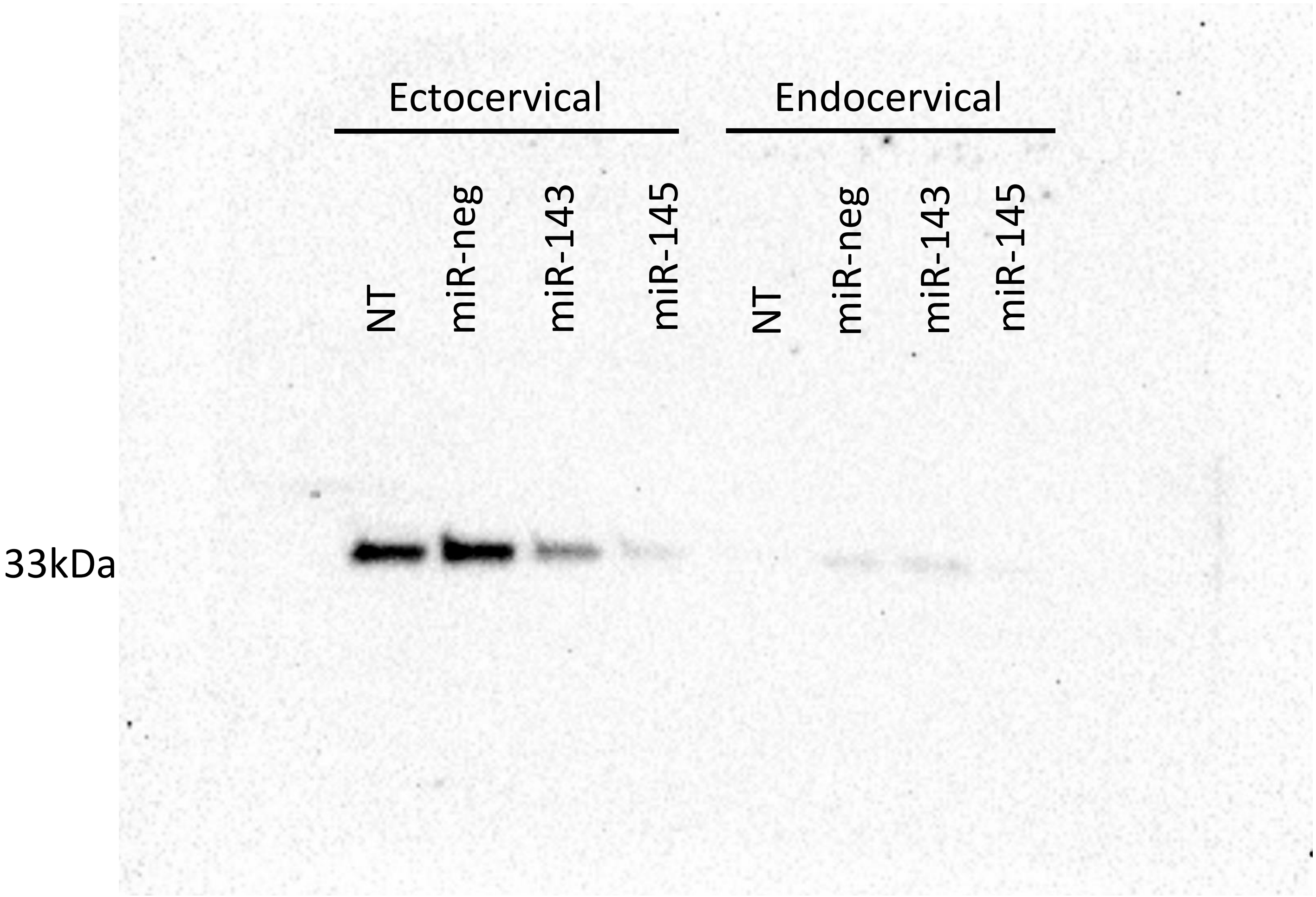

CCND2 Beta Actin, exposure 1 minute

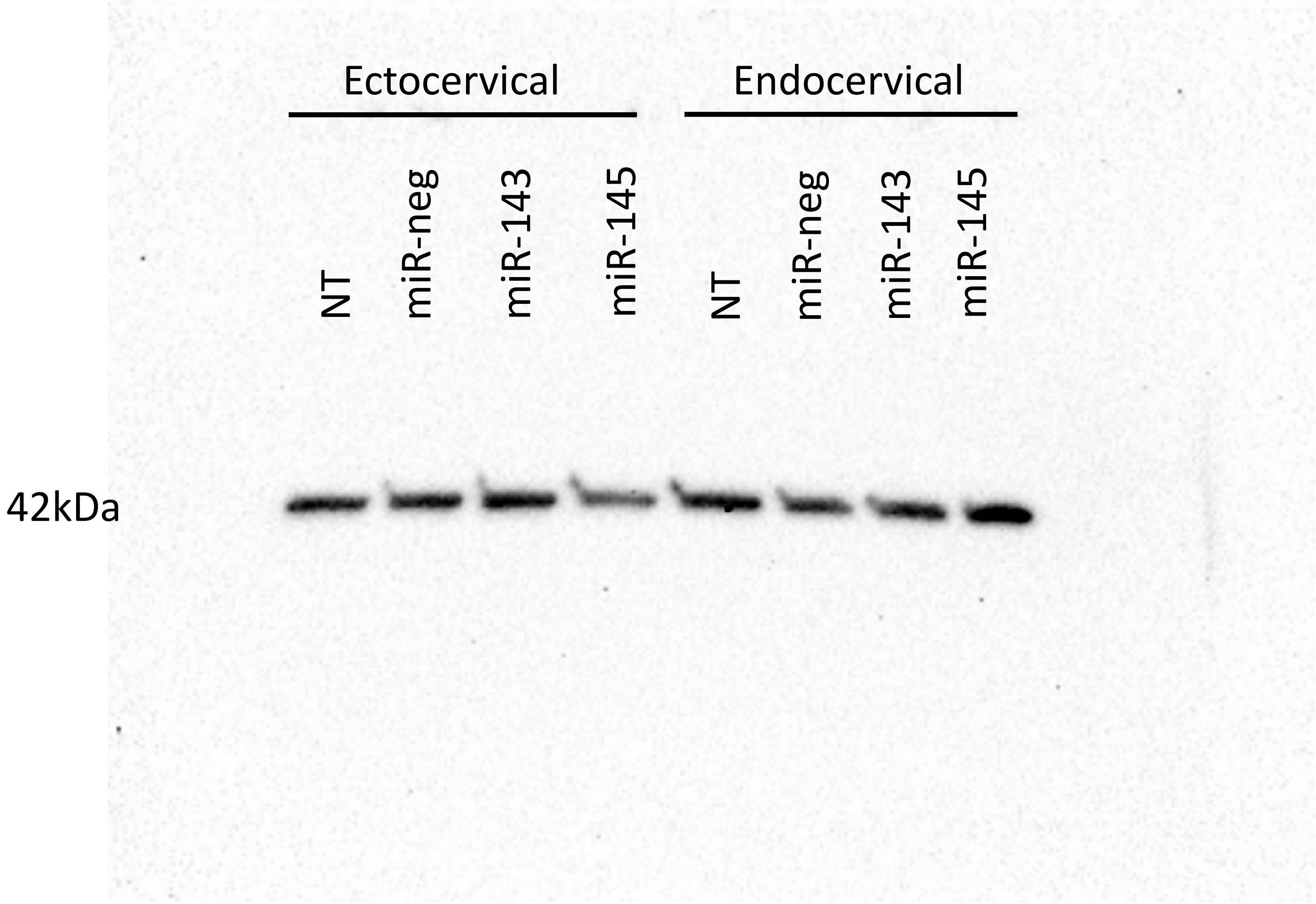

Supplement: Supplementary file 1 — Supplemental info and data [file 41598_2017_3217_MOESM1_ESM.pdf]
